# *THSD7A* Exacerbates Atherosclerosis via Activation of Signaling Axis *αvβ3/CEBPD/IL1A*

**DOI:** 10.1002/advs.77422

**Published:** 2026-08-24

**Authors:** Jiankun Liu, Hongfei Liu, Yiqi Wan, Andong Wu, Jiayu Qiu, Xueting Gong, Ya Zhao, Yuanyuan Li, Bingbing Zhou, Yangyi Zheng, Fang Wu, Yuanzheng Zhu, Weixin Lv, Xueer Li, Qiang Yuan, Xiao‐Li Tian

**Affiliations:** ^1^ Aging and Vascular Diseases Human Aging Research Institute and School of Life Science Nanchang University Jiangxi Key Laboratory of Aging and Diseases Nanchang China; ^2^ Vascular Function Laboratory Human Aging Research Institute and School of Life Science Nanchang University Jiangxi Key Laboratory of Human Aging Nanchang China

**Keywords:** atherosclerosis, CESBPD, endothelial inflammation, THSD7A, αvβ3

## Abstract

Our previous genome‐wide screening has linked *THSD7A* (thrombospondin type 1 domain containing 7A) gene to human coronary artery disease (CAD). How *THSD7A* contributes to atherosclerosis, however, remains unclear. In this study, we show THSD7A is increased in human carotid arteries and mouse atherosclerotic plaques. In apolipoprotein E knockout (*ApoE^−/−^*) mice, the overexpression of *THSD7A* increases monocyte‐endothelial adhesion and macrophage infiltration, exacerbating atherosclerotic lesions, whereas *Thsd7A* knockout (*Thsd7A^−/−^*) attenuates these phenotypic changes. Integrated single‐cell and bulk transcriptomic profiles demonstrate that *THSD7A* activates *IL1A* (Interleukin 1 alpha) signaling, augmenting endothelial inflammation. Mechanistically, THSD7A binds to integrin αvβ3, and such binding activates ERK (Extracellular signal‐regulated kinase) and augments *IL1A*‐associated proinflammatory signaling. Notably, *IL1A* serves as a transcriptional target of *CEBPD* (CCAAT/enhancer‐binding protein delta), and *CEBPD* knockdown rescues *THSD7A‐*mediated endothelial inflammation. These findings establish *THSD7A* as a novel and critical mediator in the regulation of the *αvβ3/CEBPD/IL1A* axis that controls endothelial inflammatory responses associated with atherosclerosis and demonstrate the importance of *CEBPD* as a downstream molecule in mediating *THSD7A*‐associated signaling. This study positions *THSD7A* as both a genetic marker and a potential therapeutic target.

## Graphical abstract

*THSD7A* exerts pro‐inflammatory effects and exacerbates atherosclerosis. Mechanistically, *THSD7A* regulates endothelial cell inflammation and atherosclerosis by activating the *αvβ3/CEBPD/IL1A* signaling axis. *THSD7A* is not only a genetic marker but also a potential therapeutic target for coronary artery disease (CAD).

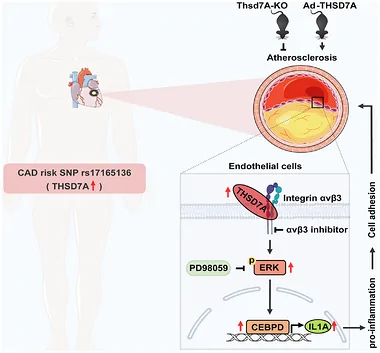

## 1 Introduction

Atherosclerosis is the hallmark pathological feature of human coronary artery disease (CAD) that is long been a major cause of mortality worldwide [1, 2, 3]. Tight junctions between endothelial cells form the barrier of the vascular wall, and endothelial cell inflammation and dysfunction are crucial for the initiation and progression of atherosclerosis [4, 5]. Inflammation induces endothelial cell injury, promotes the expression of various adhesion molecules, and thereby recruits circulating leukocytes to bind to activated endothelial cells. These early biological processes initiate the formation of atherosclerotic lesions and facilitate their progression [6, 7, 8, 9]. Genetic predisposition a fundamental factor in CAD, contributing 40% to 60% of its pathogenesis. Therefore, a deeper understanding of the genetic mechanisms underlying CAD may lead to the development of novel precision therapeutic strategies [1, 10, 11, 12, 13, 14, 15, 16, 17, 18].

Although numerous differentially expressed genes have been identified in the pathological process of atherosclerosis, their genetic association with CAD remains to be determined. Our research group conducted the first genome‐wide association study (GWAS) of CAD in the Han Chinese population, and identified that single nucleotide polymorphism (SNP) rs17165136 in *THSD7A* (thrombospondin type 1 domain containing 7A) is associated with CAD. The protective allele of this SNP is correlated with downregulated expression of its host gene, *THSD7A* [19]. *THSD7A* is a transmembrane protein preferentially expressed in the human placental vasculature and umbilical vein endothelial cells. It plays an important role in regulating cell proliferation, migration, and angiogenesis [20, 21, 22, 23, 24]. We reported that *THSD7A* knockdown in monocytes regulates the expression of *ICAM1* (Intercellular adhesion molecule 1), *L‐selectin*, and *integrin beta 2*. It functionally participates in cell adhesion [19]. A previous family based genome‐wide linkage scan revealed that the *THSD7A* gene is associated with venous thromboembolism, and endothelial dysfunction constitutes a common pathological basis for venous thrombosis and atherosclerosis [25, 26]. The proangiogenic effect of *THSD7A* may promote the progression of atherosclerotic diseases and destabilize plaques [27]. Furthermore, the expression level of *THSD7A* is higher in unstable plaques compared with stable plaques in CAD patients [28]. These findings indicate that *THSD7A* is functionally involved in atherosclerosis and CAD processes. However, the exact role and mechanism of *THSD7A* in vascular endothelial homeostasis and atherosclerosis remain unclear.

Herein, we attempted to evaluate the contribution of *THSD7A* to atherosclerosis using adenovirus mediated *THSD7A* overexpressing mice and *Thsd7A^−/−^* mice and investigated the underlying mechanisms.

## 2 Results

### 2.1 THSD7A Expression Is Elevated in Human Carotid Plaques and in Plaques of ApoE‐Deficient Mice, and Its Overexpression Exacerbates Atherosclerotic Lesions

First, we determined the contribution of *THSD7A* in atherosclerotic lesions. Analysis of published microarray data from human carotid plaques (GEO accession number: GSE43292) revealed an elevated expression level of *THSD7A* (Figure 1A). Another set of patient data from GSE120521 also demonstrated that the expression of *THSD7A* was higher in unstable plaques compared with stable plaques (Figure 1B). When compared with C57BL/6J wild‐type (WT) mice, both *THSD7A* mRNA and protein were highly expressed in the aorta of *ApoE^−/−^* mice fed a high‐fat diet for 6 weeks under the same conditions (Figure 1C–E). Subsequently, we performed double immunofluorescence staining for THSD7A and the endothelial cell marker CD31 (Cluster of Differentiation 31). The results showed that THSD7A was expressed in arteries without lesions, whereas its expression was increased in the aortic root and thoracic aorta in atherosclerotic lesions (Figure 1F).

**FIGURE 1:**
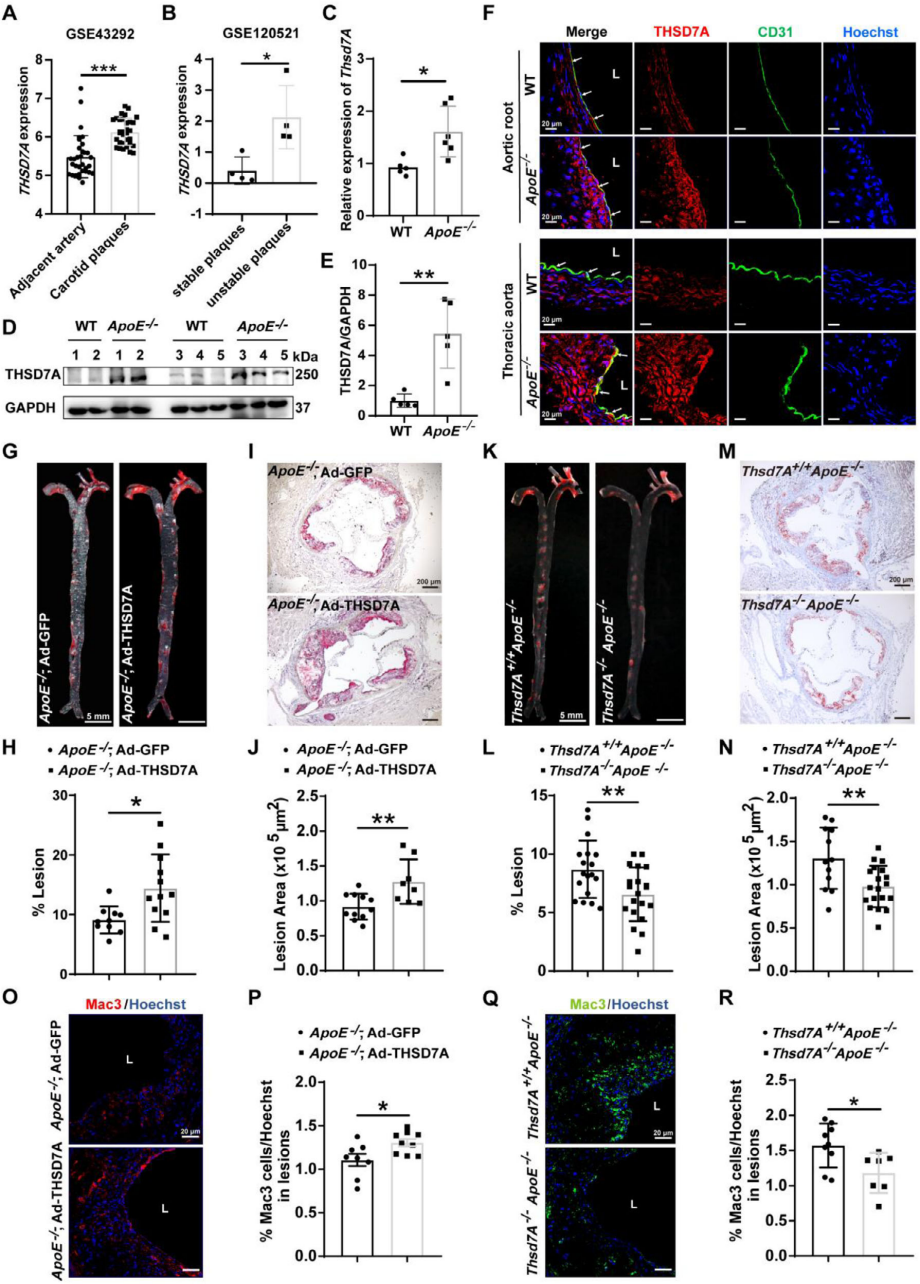
*THSD7A* is up‐regulated in human carotid plaques and ApoE‐deficient mouse plaques, and overexpression of the *THSD7A* exacerbates atherosclerotic lesions. (A) Quantitative analysis of *THSD7A* mRNA expression showing its upregulation in human carotid plaques compared with matched adjacent vascular tissues (*n* = 32 per group) (public microarray dataset GSE43292). (B) Data from GSE120521 patients demonstrating increased *THSD7A* expression in unstable plaques relative to stable plaques (*n* = 4 per group). (C) *THSD7A* mRNA expression in the whole aortic tree of WT mice and *ApoE^−/−^* mice fed a high‐fat diet for 6 weeks, detected by qPCR (WT, and *ApoE^−/−^*, *n* = 5–6 mice per group). (D‐E) Quantitative analysis of THSD7A protein expression in the whole aortic tree of WT mice and *ApoE^−/−^* mice (*n* = 5 mice per group). (F) Immunofluorescence staining of blood vessels in different regions of WT mice and *ApoE^−/−^* mice. Representative images show double immunofluorescence staining of THSD7A (red) and CD31 (endothelial cells, green) in the aortic root and thoracic aorta of mice (*n* = 3 mice per group). Scale bars: 20 µm. (G‐J) Representative images and quantitative analysis of Oil Red O stained atherosclerotic lesions in the en face aorta (Ad‐GFP, and Ad‐THSD7A, *n* = 10–12 mice per group) and aortic root (Ad‐GFP, and Ad‐THSD7A, *n* = 8‐11 mice per group) of mice after *THSD7A* overexpression. Scale bars: 5 mm (for en face aorta); 200 µm (for aortic root). (K–N) Representative images and quantitative analysis of Oil Red O stained atherosclerotic lesions in the en face aorta (*Thsd7A^+^* ^/+^ *ApoE^−/−^*, and *Thsd7A^−/−^ApoE^−/−^*, *n* = 18–20 mice per group) and aortic root (*Thsd7A^+^* ^/+^ *ApoE^−/−^*, and *Thsd7A^−/−^ApoE^−/−^*, *n* = 12–19 mice per group) of mice with *Thsd7A* gene knockout. Scale bars: 5 mm (for en face aorta); 200 µm (for aortic root). (O,P) Representative images and quantitative analysis of macrophage infiltration in mice with *THSD7A* gain‐of‐function (Ad‐GFP, and Ad‐THSD7A, *n* = 8–9 mice per group). Hoechst (nuclei, blue); Mac3 (macrophages, red). Scale bars: 20 µm. (Q,R) Representative images and quantitative analysis of macrophage infiltration in mice with *Thsd7A* loss‐of‐function (*Thsd7A^+^* ^/+^ *ApoE^−/−^*, and *Thsd7A^−/−^ApoE^−/−^*, *n* = 7–9 mice per group). Hoechst (nuclei, blue); Mac3 (macrophages, green). Scale bars: 20 µm. A‐C, E, H, J, L, N, P, R: Data are expressed as mean ± standard error of the mean (SEM). Unpaired two‐tailed Student's *t*‐tests were performed. ^*^ *p* < 0.05, ^**^ *p* < 0.01, ^***^ *p* < 0.001.

To explore the direct involvement of *THSD7A* in the process of atherosclerosis, 8‐week‐old mice were placed on a high‐fat diet for 6 weeks, and the aorta and aortic root were excised and stained with Oil Red O to visualize atherosclerotic plaques. Localization of distinct GFP green fluorescence in blood vessels was seen in tail vein‐injected adenoviral *ApoE^−/−^* mice, and immunofluorescence results showed successful overexpression of Ad‐THSD7A in blood vessels (Figure S1A,B). En face analysis showed a 58.4% increase in atherosclerotic lesion area in the aortas of *ApoE^−/−^*; Ad‐THSD7A mice compared with those of control *ApoE^−/−^*; Ad‐GFP mice (Figure 1G,H); quantitative analysis of cross‐sectional lesion area of the aortic root showed a 39.06% increase in lesion area in the aortas of *ApoE^−/−^*; Ad‐THSD7A mice (Figure 1I,J). Meanwhile, we generated global *Thsd7A^−/−^ApoE^−/−^* double knockout mice on an *ApoE^−/−^* hyperlipidemic mouse background. Compared with littermate *Thsd7A^+/+^ApoE^−/−^* mice, aortic atherosclerotic lesion area was reduced by 24.59% in *Thsd7A^−/−^ApoE^−/−^* mice (Figure 1K,L) and aortic root lesions were reduced by 25.01% (Figure 1M,N). Considering that the beneficial effects of global Thsd7A knockout might stem from contributions by other cell types, we generated an adeno‐associated virus (AAV9‐EC‐shThsd7A) driven by the *ICAM2* promoter to specifically knockdown *Thsd7A* in vascular endothelial cells. The results showed that downregulation of *Thsd7A* expression in endothelial cells reduced the formation of atherosclerotic plaques (Figure S2A–G). Notably, *Thsd7A* deficiency does not affect blood glucose, blood pressure, or blood lipids in mice (Table 1). Additionally, in *ApoE^−/−^* mice with gain‐of‐function or loss‐of‐function *THSD7A*, macrophage infiltration increased or decreased accordingly (Figure 1O–R), which is also an indicator of atherosclerosis progression. Overall, overexpression of *THSD7A* exacerbated atherosclerotic lesions.

**TABLE 1:** Characterization of blood glucose, blood pressure and lipids in *Thsd7A^+^* ^/+^ *ApoE^−/−^* and *Thsd7A^−/−^ApoE^−/−^* mice fed with high‐fat diets.

Group	*Thsd7A^+^* ^/+^ *ApoE^−/−^*	*Thsd7A^−/−^ApoE^−/−^*	*p*‐value
Blood pressure (mmHg)	140.2 ± 5.418 (*n*=9)	135 ± 4.83 (*n*=9)	*p* =0.4822
Blood glucose (mmol/L)	6.874 ± 0.2996 (*n*=39)	6.36 ± 0.223 (*n*=40)	*p*=0.1710
TC (mmol/L)	31.17 ± 1.093 (*n*=44)	32.83 ± 1.426 (*n*=52)	*p*=0.3699
TG (mmol/L)	3.141 ± 0.2078 (*n*=44)	3.768 ± 0.2543 (*n*=52)	*p*=0.0654
HDL‐C (mmol/L)	1.881 ± 0.0425 (*n*=44)	1.786 ± 0.0441 (*n*=52)	*p*=0.1308
LDL‐C (mmol/L)	12.31 ± 0.5188 (*n*=44)	10.73 ± 0.7620 (*n*=52)	*p*=0.0999

Blood pressure and blood biochemical parameters were measured in mice. All mice were anesthetized via intraperitoneal injection of sodium pentobarbital (50 mg/kg), followed by blood pressure (*Thsd7A^+^* ^/+^ *ApoE^−/−^*, and *Thsd7A^−/−^ApoE^−/−^*, *n* = 9 mice per group) and blood glucose (*Thsd7A^+^* ^/+^ *ApoE^−/−^*, and *Thsd7A^−/−^ApoE^−/−^*, *n* = 39–40 mice per group) measurements. Serum was obtained by centrifuging mouse blood for lipid profile analysis (TC, TG, HDL‐C, LDL‐C, *Thsd7A^+^* ^/+^ *ApoE^−/−^*, and *Thsd7A^−/−^ApoE^−/−^*, *n* = 44–52 mice per group). Data are expressed as mean ± SEM. Unpaired two‐tailed Student's *t*‐tests were performed. *p* > 0.05, no significant.

### 2.2 Genetic Overexpression of THSD7A Promotes Endothelial Cell Inflammation

Atherosclerosis is a chronic inflammatory disease. To identify the specific biological processes in which THSD7A is involved in atherosclerosis, we analyzed single‐cell sequencing data from carotid plaques obtained from patients. Through cell clustering and type annotation, we assessed the normalized average expression levels of THSD7A in different cell types (Figure S3A,B). Our results show that THSD7A is highly expressed and localized primarily in endothelial cells, and that its expression levels are elevated in endothelial cells from patients’ carotid plaques (Figure 2A–C). Inflammation of vascular endothelial cells contributes to accelerating the initiation and persistence of atherosclerotic plaques, and monocyte adhesion to endothelial cells is an early hallmark of atherosclerosis [6, 7, 8, 9]. We investigated the role of *THSD7A* in endothelial cell inflammation. Through adenovirus mediated overexpression of *THSD7A* in vivo, we observed a significant increase in monocyte‐endothelial cell adhesion isolated from *THSD7A* overexpressing *ApoE^−/−^* mice compared with the Ad‐GFP group (Figure 2D). The bright‐field images show that the endothelial cells have all grown into a fused monolayer and exhibit a uniform, consistent monolayer morphology across different fields of view. The cell density and growth status between the two groups are highly comparable (Figure S4). Furthermore, adhesion was significantly decreased in *Thsd7A* deficient *ApoE^−/−^* mice compared with littermate controls (*Thsd7A^+^* ^/+^ *ApoE^−/−^*) (Figure 2E). Similar results were obtained in human umbilical vein endothelial cells (HUVECs) and human coronary artery endothelial cells (HCAECs) cultured in vitro. Following adenoviral transfection of the cells, no drug screening was required; fluorescence microscopy revealed a GFP infection efficiency of 70%–95%, and Western blot analysis confirmed significant overexpression of THSD7A (Figure S5A–D). When THSD7A was overexpressed (MOI = 10), the adhesion of green BCECF‐AM–labeled monocytes to endothelial cells increased (Figure 2F,G,L). The bright‐field images show a uniform, dense, and fused monolayer of endothelial cells, indicating that the two groups are well comparable (Figure S6A,B). We used tumor necrosis factor‐α (TNF‐α) and oxidized low‐density lipoprotein (ox‐LDL) to establish endothelial inflammation models, respectively. We first evaluated the effect of high concentrations of ox‐LDL on endothelial cell apoptosis. The results showed that 100 µg/mL ox‐LDL slightly increased endothelial cell apoptosis (Figure S7A, B), but its primary effect was still to induce endothelial cell inflammation (Figure S8A–G). knockdown of THSD7A reduced the adhesion of monocytes to endothelial cells induced by TNF‐α and ox‐LDL. (Figure 2H–K, M–P). It is worth noting that we must acknowledge that GFP fluorescence may interfere with the green‐labeled monocytes during cell adhesion; however, this effect is negligible when using adenovirus (MOI = 10). However, the decision not to employ a dual‐fluorescent labeling strategy in this study presents a limitation, as it precludes the direct observation and simultaneous tracking of both endothelial cells and monocyte populations in the co‐culture system. Future studies that employ different fluorescent markers for each cell type will help to more precisely elucidate the specific contributions of each cell type to the observed phenotypes.

**FIGURE 2:**
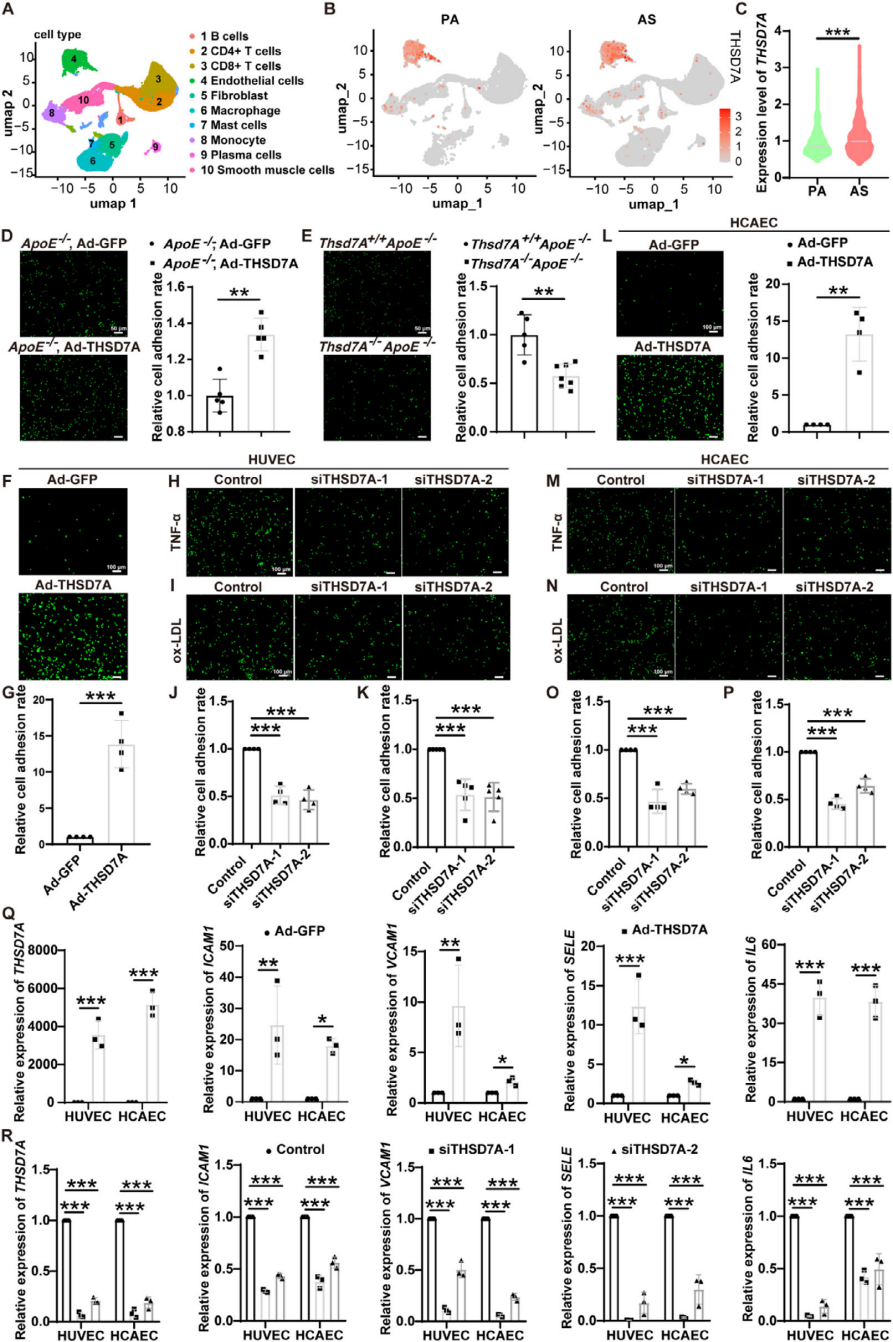
Overexpression of *THSD7A* promotes monocyte‐endothelial cell adhesion and the expression of endothelial adhesion molecules and proinflammatory cytokines. (A–C) UMAP plots of single cells from carotid plaques of patients with atherosclerosis, with 9 major cell types labeled in different colors. *THSD7A* is predominantly highly expressed in endothelial cells of atherosclerotic plaques. Violin plot showing the expression level changes of *THSD7A* in endothelial cells of carotid plaques. PA: patient‐matched proximal adjacent, and AS: atherosclerotic plaque (*n* = 3 per group). (D) Representative images of monocyte‐endothelial cell adhesion in mice with in vivo *THSD7A* overexpression. Monocytes and endothelial cells were isolated from *ApoE^−/−^* mice injected with Ad‐THSD7A and Ad‐GFP via the tail vein, respectively (*n* = 5 mice per group). Scale bars: 50 µm. (E) Representative images and quantification of cell adhesion in *Thsd7A* deficient mice (*Thsd7A^+^* ^/+^ *ApoE^−/−^*, and *Thsd7A^−/−^ApoE^−/−^*, *n* = 5–7 mice per group). Scale bars: 50 µm. (F,G,L) Representative images and quantification of monocyte‐endothelial cell adhesion after *THSD7A* overexpression in HUVECs and HCAECs, respectively (*n* = 4 independent experiments). Scale bars: 100 µm. (H–K) Representative images of cell adhesion in HUVECs with *THSD7A* knockdown. Cells were treated with TNF‐α (10 ng/mL) and ox‐LDL (100 µg/mL) for 24 h. Quantitative data are shown in the lower panel (TNF‐α group, *n* = 4 independent experiments; ox‐LDL group, *n* = 5 independent experiments). Scale bars: 100 µm. (M–P) Representative images of cell adhesion in HCAECs with *THSD7A* knockdown. Cells were treated with TNF‐α (10 ng/mL) and ox‐LDL (100 µg/mL) for 24 h. Quantitative data are shown in the lower panel (*n* = 4 independent experiments). Scale bars: 100 µm. (Q) Expression levels of adhesion molecules (*ICAM1*, *VCAM1*, and *SELE*) and inflammatory factor (*IL6*) detected by qPCR in HUVECs and HCAECs with *THSD7A* overexpression (*n* = 3 independent experiments). (R) mRNA expression levels of *ICAM1*, *VCAM1*, *SELE*, and *IL6* in HUVECs and HCAECs with *THSD7A* knockdown after treatment with TNF‐α (10 ng/mL) for 24 h (*n* = 3 independent experiments). Panels D, E, F, H, I, L, M and N, green: BCECF‐AM‐labeled monocytes. For panels C, D, E, G, and L, data are expressed as mean ± SEM. Unpaired two‐tailed Student's *t*‐test. ^**^ *p* < 0.01, ^***^ *p* < 0.001. For panels J, K, O, and P, values are expressed as mean ± SEM. One‐way ANOVA. ^***^ *p* < 0.001. For panels Q and R, values are expressed as mean ± SEM. Two‐way ANOVA. ^*^ *p* < 0.05, ^**^ *p* < 0.01, ^***^ *p* < 0.001.

To further elucidate how *THSD7A* mediates atherosclerosis, we performed gain or loss of function assays of *THSD7A* in endothelial cells, and measured the expression of adhesion molecules and proinflammatory cytokines‐classic hallmarks of the endothelial inflammatory response. We found that *THSD7A* overexpression in HUVECs and HCAECs increased the expression of adhesion molecules *ICAM1*, *VCAM1* (Vascular cell adhesion molecule 1), and *SELE* (Selectin E) and the proinflammatory cytokine *IL6* (Interleukin 6) (Figure 2Q). Under TNF‐α stimulation, siRNA mediated *THSD7A* knockdown reduced the expression of *ICAM1*, *VCAM1*, *SELE*, and *IL6* compared with the control group (Figure 2R). These results suggest that *THSD7A* participates in the initiation of atherosclerosis by promoting the endothelial inflammatory response, thereby inducing monocyte‐endothelial cell adhesion and upregulating the expression of adhesion molecules and proinflammatory cytokines.

### 2.3 THSD7A Activates Downstream IL1A Signaling to Promote Endothelial Cell Inflammation

To investigate the mechanism by which *THSD7A* regulates endothelial cell inflammation, we performed RNA sequencing on HUVECs after *THSD7A* overexpression or knockdown. Gene Set Enrichment Analysis (GSEA) revealed that both *THSD7A* overexpression and knockdown are involved in a common biological process‐the acute inflammatory response (Figure 3A), which is closely associated with atherosclerosis. To further validate differential co‐expression between genes and *THSD7A*, we integrated the acute inflammatory response processes from these two datasets, including 25 enriched genes in *THSD7A* overexpressing cells and 27 enriched genes in *THSD7A* knockdown cells. These two gene sets overlapped by 10 genes, and under the threshold of FDR < 0.05, 4 genes (*IL31RA*, *IL1A*, *IL6*, *ICAM1*) were identified as differentially co‐expressed with *THSD7A* (Figure 3B–D), representing potential downstream targets. We then validated the co‐expression relationship between these 4 genes and *THSD7A* in HUVECs and HCAECs. Notably, *IL1A* exhibited the most significant differential change (Figure 3E,F). Subsequently, we characterized the involvement of *IL1A* in inflammatory responses. We performed *IL1A* loss‐of‐function in endothelial cells and detected the expression of adhesion molecules, pro‐inflammatory cytokines, and monocyte‐endothelial cell adhesion. Results showed that *IL1A* knockdown reduced the expression of *ICAM1*, *VCAM1*, *SELE*, and *IL6* in HUVECs and HCAECs under TNF‐α stimulation (Figure 3G). For cellular functions, decreased *IL1A* expression in endothelial cells reduced cell adhesion under TNF‐α and ox‐LDL stimulation (Figure 3H,I). These findings indicate that *THSD7A* activates downstream *IL1A* signaling to promote endothelial cell inflammation.

**FIGURE 3:**
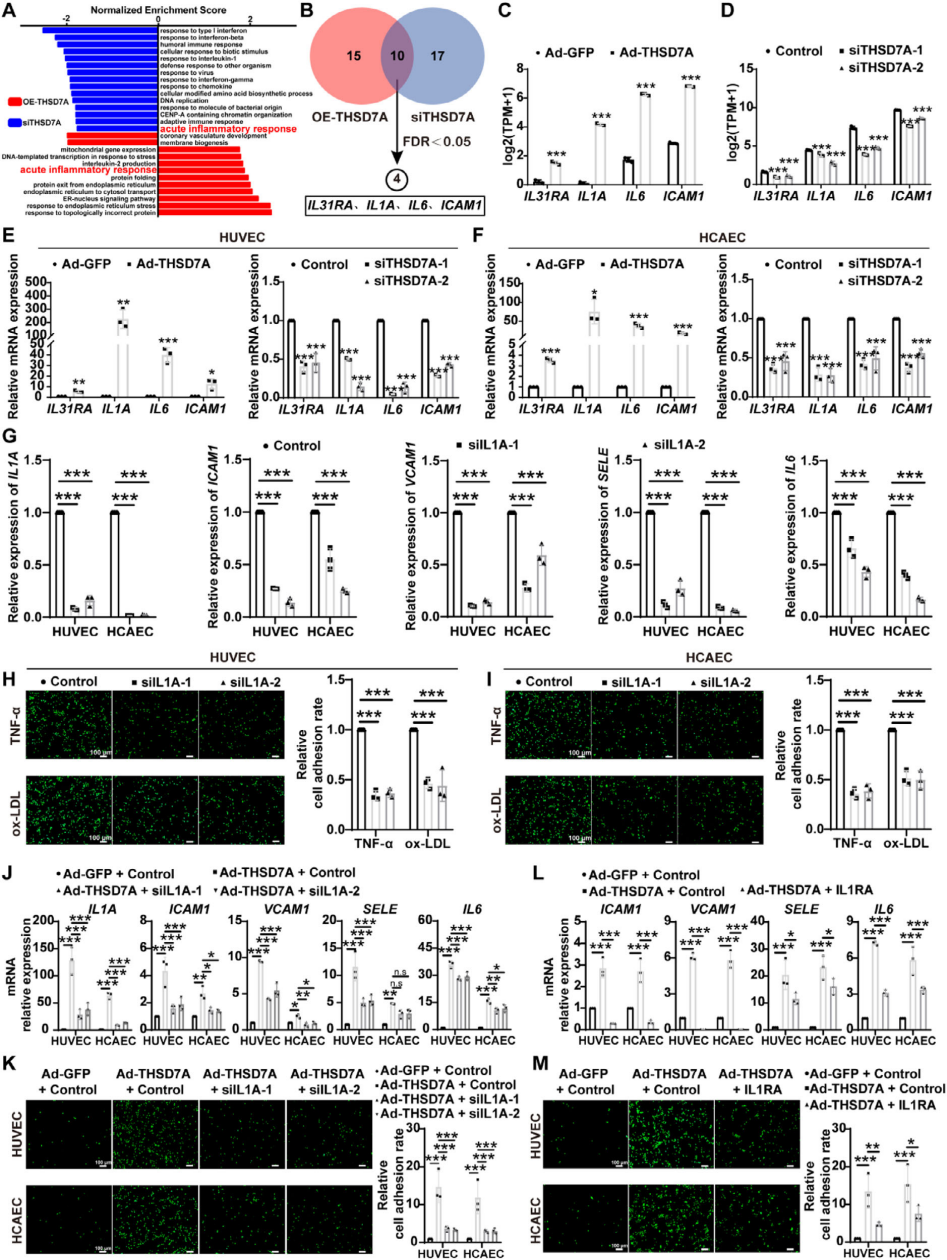
*THSD7A* promotes inflammatory responses in endothelial cells through *IL1A* signaling. (A) Gene Ontology (GO) analysis of HUVECs with *THSD7A* overexpression (*n* = 3 independent experiments) or knockdown (*n* = 3 independent experiments), illustrating the biological processes involved. (B) Venn diagram depicting genes associated with the acute inflammatory response in two datasets (*THSD7A* overexpression and *THSD7A* knockdown, *n* = 3 independent experiments). Under the threshold of FDR < 0.05, 4 genes (*IL31RA*, *IL1A*, *IL6*, *ICAM1*) were identified (*n* = 3 independent experiments). (C‐D) Expression levels of *IL31RA*, *IL1A*, *IL6*, and *ICAM1* from RNA‐seq data (*n* = 3 independent experiments). (E‐F) Validation of the 4 candidate genes by qPCR in HUVECs and HCAECs, respectively; *IL1A* showed the most significant differential expression (*n* = 3 independent experiments). (G) mRNA expression levels of *IL1A*, *ICAM1*, *VCAM1*, *SELE*, and *IL6* in HUVECs and HCAECs with *IL1A* knockdown, following treatment with TNF‐α (10 ng/mL) for 24 h (*n* = 3 independent experiments). (H) Representative images of cell adhesion in HUVECs with *IL1A* knockdown. Quantitative data were obtained after treatment with TNF‐α (10 ng/mL) or ox‐LDL (100 µg/mL) for 24 h (*n* = 3 independent experiments). Scale bars: 100 µm. (I) Representative images of cell adhesion in HCAECs with *IL1A* knockdown. Quantitative data were obtained after treatment with TNF‐α (10 ng/mL) and ox‐LDL (100 µg/mL) for 24 h (*n* = 3 independent experiments). Scale bars: 100 µm. (J) HUVECs or HCAECs were infected with Ad‐THSD7A for 48 h, followed by treatment with two *IL1A*‐targeting siRNAs for 24 h. mRNA expression levels of *IL1A*, *ICAM1*, *VCAM1*, *SELE*, and *IL6* were detected by qPCR (*n* = 3 independent experiments). (K) Representative images and quantification of cell adhesion in HUVECs or HCAECs with *THSD7A* overexpression followed by *IL1A* knockdown (*n* = 3 independent experiments). Scale bars: 100 µm. (L) mRNA expression levels of *ICAM1*, *VCAM1*, *SELE*, and *IL6* quantified by qPCR in HUVECs or HCAECs with *THSD7A* overexpression, following treatment with IL1RA (10 ng/mL) for 24 h (*n* = 3 independent experiments). (M) Representative images and quantification of cell adhesion in HUVECs or HCAECs with *THSD7A* overexpression, following treatment with IL1RA (10 ng/mL) for 24 h (*n* = 3 independent experiments). Scale bars: 100 µm. For panels C, D, E, F, H, I, J, K, L, and M, values are expressed as mean ± (SEM). Two‐way ANOVA. ^*^ *p* < 0.05, ^**^ *p* < 0.01, ^***^ *p* < 0.001.

Next, we queried whether inhibiting *IL1A* could alleviate *THSD7A* overexpression mediated endothelial inflammation. *IL1A* has been shown to exacerbate atherosclerotic progression via its receptor *IL1R1* [29]. We performed *IL1A* loss‐of‐function using siRNA interference or an *IL1A* receptor antagonist (IL1RA) and examined its impact on *THSD7A* promoted endothelial inflammation, including the expression of molecular markers (adhesion molecules and pro‐inflammatory cytokines) and cell adhesion. In HUVECs and HCAECs overexpressing *THSD7A*, Ad‐THSD7A infection significantly increased the expression of *IL1A*, *ICAM1*, *VCAM1*, *SELE*, and *IL6*, as well as cell adhesion, compared with Ad‐GFP. However, these effects of *THSD7A* overexpression were rescued by siRNA interference (Figure 3J,K). Similarly, after IL1RA intervention, *THSD7A* overexpression induced upregulation of adhesion molecules (*ICAM1*, *VCAM1*, *SELE*), pro‐inflammatory cytokine (*IL6*), and cell adhesion were effectively rescued (Figure 3L,M). Thus, these data demonstrate that inhibiting *IL1A* signaling can effectively reduce *THSD7A* promoted endothelial cell inflammation.

### 2.4 Integrin αvβ3 Is Crucial for THSD7A to Promote Endothelial Cell Inflammation and Atherosclerosis

Subsequently, we determined the contribution of THSD7A interacting proteins to endothelial inflammation and atherosclerosis. Integrin αvβ3, a key cell adhesion molecule composed of an αv subunit and a β3 subunit, is a membrane protein mediating intracellular and extracellular signal transduction. It has been demonstrated that the co‐localization of integrin αvβ3 with THSD7A regulates angiogenesis [21]. We found that THSD7A interacts with αvβ3 in resting endothelial cells (Figure 4A,C). Further immunofluorescence and co‐immunoprecipitation experiments with overexpressed THSD7A also revealed a robust interaction between the two proteins (Figure 4B, D–H). Next, we investigated the role of αvβ3 in THSD7A promoted endothelial inflammation. HUVECs or HCAECs infected with Ad‐THSD7A exhibited significantly increased expression of inflammatory markers (*IL1A*, *ICAM1*, *VCAM1*, *SELE*, and *IL6*), whereas knockdown of *αvβ3* reduced the expression of these inflammatory markers (Figure 4I,K). We also evaluated cell adhesion, a critical phenotypic feature of endothelial inflammation. Knockdown of *αvβ3* significantly attenuated *THSD7A* overexpression induced monocyte‐endothelial adhesion (Figure 4J,L).

**FIGURE 4:**
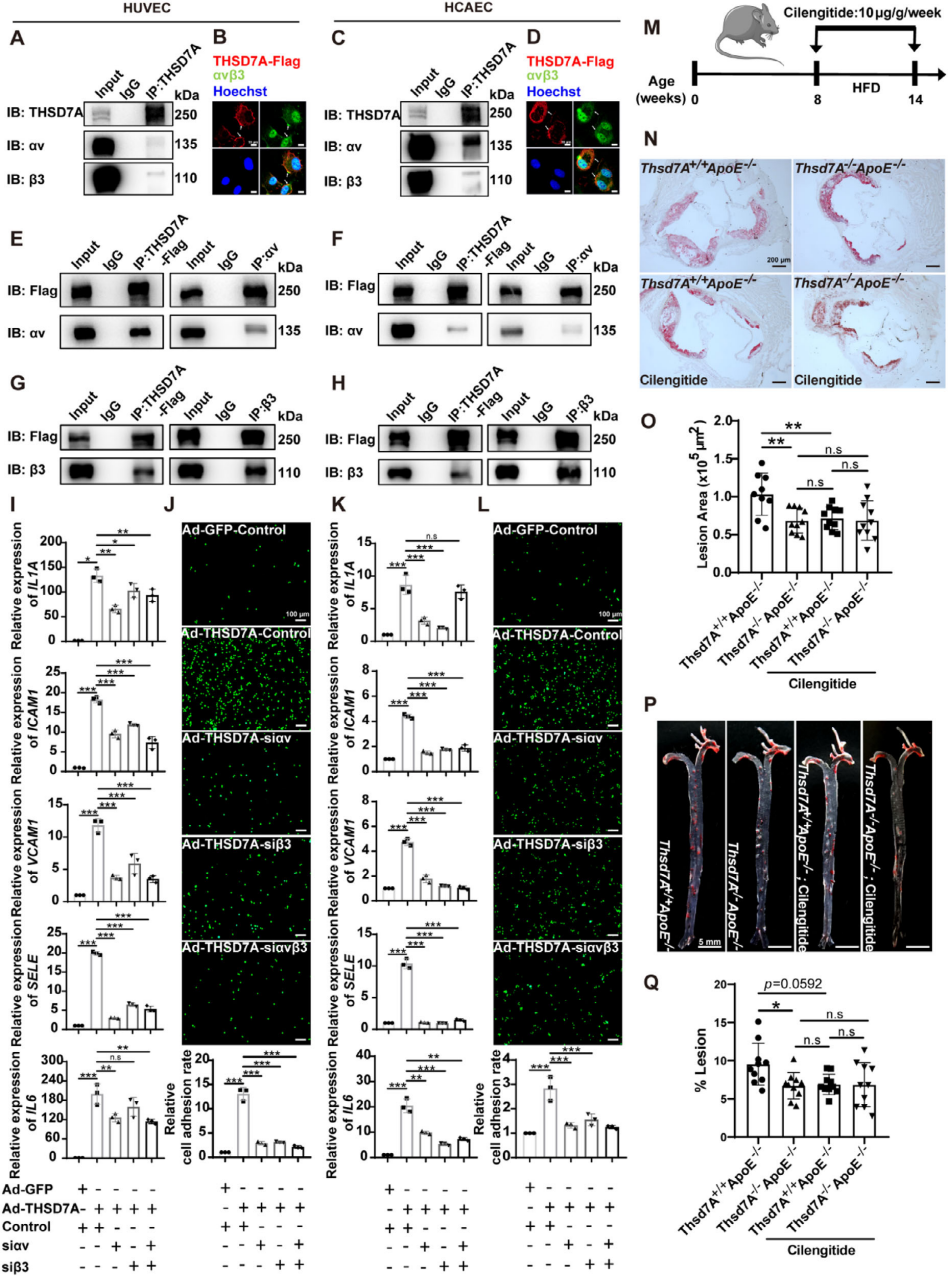
The interaction between *THSD7A* and integrin *αvβ3* mediates endothelial cell inflammation and atherosclerosis. (A, C) Co‐IP assays were conducted to assess the interaction between THSD7A and integrin αvβ3 in resting HUVECs or HCAECs. (B, D) Representative images of THSD7A and αvβ3 co‐localization on the cell membrane in HUVECs or HCAECs at 48 h after Ad‐THSD7A infection. THSD7A (red); αvβ3 (green); Hoechst (blue). Scale bars: 10 µm. (E,F) Co‐IP assays detecting the protein interaction between THSD7A and the αv subunit of integrin αvβ3 after Ad‐THSD7A overexpression. (G,H) Co‐IP assays detecting the protein interaction between THSD7A and the β3 subunit of integrin αvβ3 after Ad‐THSD7A overexpression. (I,J) mRNA expression levels of *IL1A*, *ICAM1*, *VCAM1*, *SELE*, and *IL6*, as well as representative images and quantification of cell adhesion, in HUVECs with *THSD7A* overexpression followed by *αvβ3* knockdown (*n* = 3 independent experiments). Scale bars: 100 µm. (K,L) mRNA expression levels of *IL1A*, *ICAM1*, *VCAM1*, *SELE*, and *IL6*, as well as representative images and quantification of cell adhesion, in HCAECs with *THSD7A* overexpression followed by *αvβ3* knockdown (*n* = 3 independent experiments). Scale bars: 100 µm. (M) Experimental design for intervention with the *αvβ3* inhibitor cilengitide (10 µg/g/week). (N,O) Representative images and quantitative analysis of Oil Red O stained atherosclerotic lesions in the aortic root of *ApoE^−/−^* mice with or without *Thsd7A* expression, following cilengitide administration and 6 weeks of high‐fat diet feeding (*n* = 9‐10 mice per group). Scale bars: 200 µm. (P,Q) Representative images and quantitative analysis of Oil Red O‐stained atherosclerotic lesions in the en face aorta of *ApoE^−/−^* mice with or without *Thsd7A* expression, following cilengitide administration and 6 weeks of high‐fat diet feeding (*n* = 9–10 mice per group). Scale bars: 5 mm. For panels I, J, K, L, O, and Q, values are expressed as mean ± SEM. One‐way ANOVA. ^*^ *p* < 0.05, ^**^ *p* < 0.01, ^***^ *p* < 0.001.

Herein, we constructed a mouse model for in vivo validation. *ApoE^−/−^* mice with or without *Thsd7A* expression were administered the αvβ3 signaling inhibitor cilengitide (10 µg/g/week) and fed a high‐fat diet for 6 weeks (Figure 4M). We found that cilengitide treatment reduced atherosclerotic plaque progression in *ApoE^−/−^* mice with normal *Thsd7A* expression, whereas cilengitide had no effect on atherosclerotic plaques in *Thsd7A* deficient *ApoE^−/−^* mice (Figure 4N–Q). In summary, *αvβ3* is an indispensable component in *THSD7A* mediated regulation of atherosclerosis. These in vitro and in vivo results indicate that *THSD7A* interacts with *αvβ3* to mediate endothelial inflammation and atherosclerosis.

### 2.5 Inhibition of ERK Signaling Rescues THSD7A Overexpression Promoted Endothelial Cell Inflammation

To further explore the *THSD7A/αvβ3* dependent proinflammatory effects in endothelial cells, we sought to identify the downstream molecular mechanisms through which *αvβ3* promotes endothelial inflammation. Previous studies have established that *αvβ3*, acting as a signal‐receiving protein, activates the intracellular *FAK/Ras/c‐Raf/ERK* signaling axis, which plays a pivotal regulatory role in cell survival, differentiation, and migration [30, 31]. Notably, *ERK* signaling holds significant importance in inflammatory responses; accumulating evidence indicates that *ERK* can directly participate in the function of nuclear transcription factors, thereby regulating the expression of adhesion molecules (*ICAM1*, *VCAM1*, *SELE*) and proinflammatory cytokines (*IL1*, *IL6*, *TNF‐α*, *MCP1*) [32, 33, 34]. Our results demonstrated that Ad‐THSD7A infection in HUVECs or HCAECs not only effectively increased THSD7A expression but also significantly activated p‐ERK (Figure 5A–C, K–M). Furthermore, we evaluated the impact of inhibiting ERK activation on the inflammatory response. PD98059 has been proven to be a safe and effective inhibitor of ERK signaling. Treatment with PD98059 in *THSD7A* overexpressing HUVECs or HCAECs led to a reduction in the expression of inflammation‐related molecular markers, including *IL1A*, *ICAM1*, *VCAM1*, *SELE* and *IL6* (Figure 5D–H, N–R). Concurrently, PD98059 treatment markedly attenuated the enhanced cell adhesion induced by *THSD7A* overexpression (Figure 5I,J,S,T). These data situate *ERK* activation as a critical, non‐redundant conduit linking *THSD7A/αVβ3* signaling to the endothelial inflammatory response.

**FIGURE 5:**
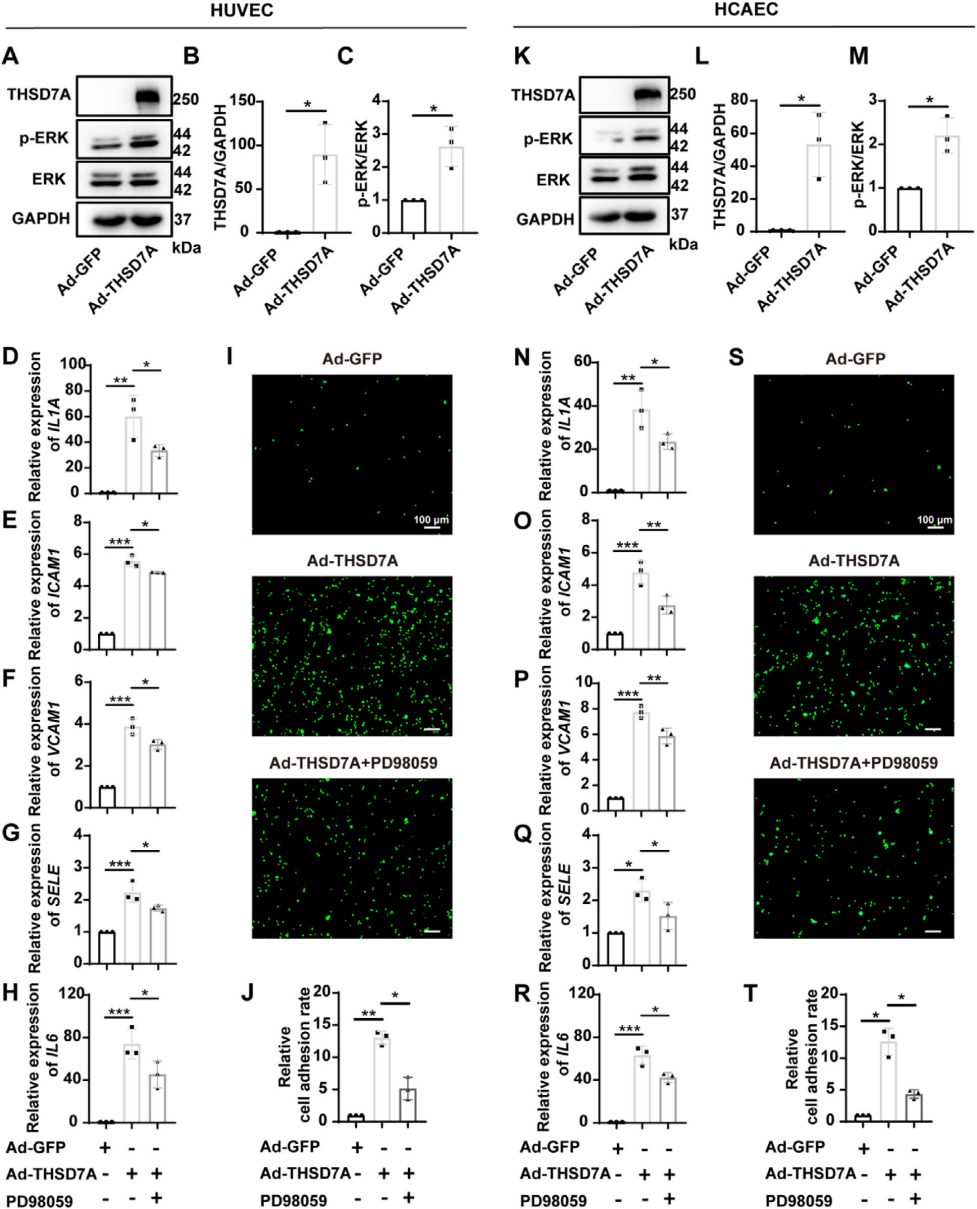
Inhibition of *ERK* signaling reduces *THSD7A* overexpression promoted endothelial cell inflammation. (A–C) Western blotting quantification and representative images of THSD7A, p‐ERK, and total ERK expression in HUVECs at 48 h after Ad‐THSD7A infection (*n* = 3 independent experiments). (D–H) mRNA expression levels of *IL1A*, *ICAM1*, *VCAM1*, *SELE*, and *IL6*‐upregulated by *THSD7A* overexpression‐were suppressed in HUVECs treated with PD98059 (final concentration: 10 µmol/L) for 24 h (*n* = 3 independent experiments). (I,J) Representative images and quantification of cell adhesion‐promoted by *THSD7A* overexpression‐were reduced in HUVECs treated with PD98059 for 24 h (*n* = 3 independent experiments). Scale bars: 100 µm. (K–M) Western blotting detection and representative images of THSD7A, p‐ERK, and total ERK expression in HCAECs with THSD7A overexpression (*n* = 3 independent experiments). (N‐R) mRNA expression levels of *IL1A*, *ICAM1*, *VCAM1*, *SELE*, and *IL6* were decreased in *THSD7A*‐overexpressing HCAECs following PD98059 treatment (*n* = 3 independent experiments). (S–T) Representative images and quantification of cell adhesion‐promoted by *THSD7A* overexpression‐were reduced in HCAECs treated with PD98059 for 24 h (*n* = 3 independent experiments). Scale bars: 100 µm. For panels B, C, L, and M, data are expressed as mean ± SEM. Student's *t*‐test. ^*^ *p* < 0.05. For panels D–H, J, N–R, and T, values are expressed as mean ± SEM. One‐way ANOVA. ^*^ *p* < 0.05, ^**^ *p* < 0.01, ^***^ *p* < 0.001.

### 2.6 THSD7A Activates the αVβ3/ERK/CEBPD Signaling Axis to Regulate IL1A Transcription and Promote Endothelial Cell Inflammation

To systematically investigate the mechanism by which the *THSD7A/αvβ3/ERK* signaling pathway promotes *IL1A*‐mediated inflammatory responses, we identified the direct regulatory factors of *IL1A*. From RNA sequencing data of *THSD7A* overexpression and knockdown, *CEBPD*, *OASL*, and *IL31RA* were screened as transcription regulation related genes. Literature retrieval confirmed that only *CEBPD* functions as a transcriptional regulator (Figure 6A). Three CEBPD recognition sites were identified in the IL1A promoter region through JASPAR database analysis (Figure 6B). ChIP‐qPCR assays confirmed that *CEBPD* binds to the *IL1A* promoter region (−1741) and regulates *IL1A* expression (Figure 6C,D). Moreover, luciferase reporter assays demonstrated reduced *IL1A* promoter activity following mutation of this *CEBPD* binding motif. These findings indicate that *CEBPD* activates *IL1A* expression via direct binding to the −1741 locus of the *IL1A* promoter region (Figure 6E). Next, we explored whether *THSD7A/αvβ3/ERK* signaling mediated activation of *IL1A* inflammatory responses is dependent on *CEBPD*. As expected, inhibition of *αvβ3* reduced *THSD7A* overexpression induced *CEBPD* upregulation (Figure 6F,G,I,J). Similarly, suppression of *ERK* signaling also decreased *CEBPD* expression promoted by *THSD7A* overexpression (Figure 6H,K).

**FIGURE 6:**
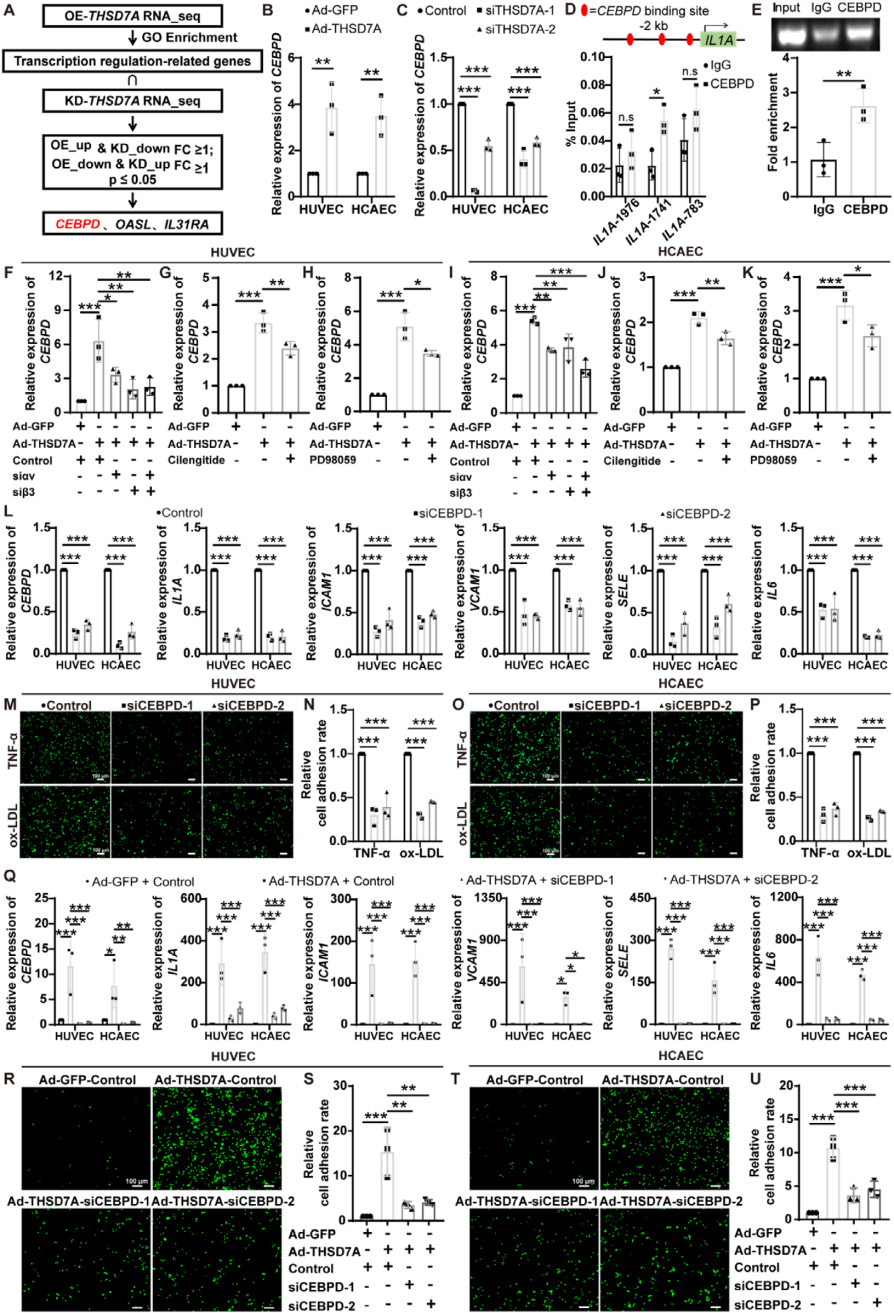
*THSD7A* activates the *αvβ3/ERK/CEBPD* signaling axis to transcriptionally regulate *IL1A* signaling and promote endothelial inflammation. (A) Identification of the transcriptional regulator *CEBPD* via screening of HUVECs with *THSD7A* overexpression or knockdown. (B–D) Sequence analysis revealed *CEBPD* binding sites within the *IL1A* gene promoter; CHIP‐qPCR confirmed direct binding of *CEBPD* to the *IL1A* promoter (*n*=3 independent experiments). (E) Luciferase reporter assays demonstrated reduced IL1A promoter activity following mutation of the CEBPD‐binding locus (*n*=3 independent experiments). (F) Detection of *CEBPD* expression following *THSD7A* overexpression and *αvβ3* knockdown in HUVECs (*n*=3 independent experiments). (G) Detection of *CEBPD* expression after adding *αvβ3* inhibitor (Cilengitide) to HUVECs overexpressing *THSD7A* (*n*=3 independent experiments). (H) Detection of *CEBPD* expression after adding *ERK* inhibitor (PD98059) to HUVECs overexpressing *THSD7A* (*n*=3 independent experiments). (I) Detection of *CEBPD* expression after *THSD7A* overexpression and *αvβ3* knockdown in HCAECs (*n*=3 independent experiments). (J) Detection of *CEBPD* expression after *THSD7A* overexpression and *αvβ3* inhibitor (Cilengitide) treatment in HCAECs (*n*=3 independent experiments). (K) Detection of *CEBPD* expression after adding *ERK* inhibitor (PD98059) to HCAECs overexpressing *THSD7A* (n=3 independent experiments). (L) Expression of *IL1A*, *ICAM1*, *VCAM1*, *SELE*, and *IL6* after *CEBPD* knockdown in HUVECs and HCAECs (*n*=3 independent experiments). (M–P) Representative images and quantification of cell adhesion in HUVECs and HCAECs after *CEBPD* knockdown (*n*=3 independent experiments). Scale bars: 100µm. (Q) Expression of *CEBPD*, *IL1A*, *ICAM1*, *VCAM1*, *SELE*, and *IL6* in HUVECs and HCAECs after *THSD7A* overexpression followed by *CEBPD* knockdown (*n*=3 independent experiments). (R‐U) Representative images and quantification of cell adhesion in HUVECs and HCAECs after *CEBPD* knockdown following *THSD7A* overexpression (*n*=3 independent experiments). Scale bars: 100µm. D, Data are expressed as mean ± SEM. Student's *t*‐test. ^**^ *p* < 0.01. E–K, S, U, Values are expressed as mean ± SEM. One‐way ANOVA. ^*^ *p* < 0.05, ^**^ *p* < 0.01, ^***^ *p* < 0.001. C, L, N, P, Q, values are expressed as mean ± SEM. Two‐way ANOVA. ^*^ *p* < 0.05, ^**^ *p* < 0.01, ^***^ *p* < 0.001.

To further clarify the central role of *CEBPD* in *THSD7A* induced activation of *IL1A* inflammatory signaling, we examined the effects of *CEBPD* intervention on inflammatory molecular markers and cell adhesion in endothelial cells. First, *CEBPD* knockdown reduced *IL1A* expression (Figure 6L), further confirming that *CEBPD* directly regulates *IL1A*. Second, we found that *CEBPD* knockdown decreased the expression of inflammation‐related markers, including *IL1A*, *ICAM1*, *VCAM1*, *SELE*, and *IL6* (Figure 6L). For cellular functions, reduced *CEBPD* expression in endothelial cells attenuated cell adhesion in TNF‐α‐ and ox‐LDL‐induced inflammatory models (Figure 6M–P). In rescue experiments, *CEBPD* knockdown diminished *THSD7A* overexpression induced upregulation of *IL1A*, as well as the expression of inflammatory markers (*ICAM1*, *VCAM1*, *SELE*, and *IL6*) (Figure 6Q). Concomitantly, decreased CEBPD expression reduced *THSD7A* overexpression promoted cell adhesion (Figure 6R–U).

Notably, *THSD7A* exists in a soluble form that exerts biological functions [35]. We found that recombinant THSD7A (rhTHSD7A) similarly increases the expression of *ICAM1*, *VCAM1*, *SELE*, and *IL6* (Figure S9A–D, I–L), enhances cell adhesion (Figure S9E,F,M,N), and upregulates *CEBPD* and *IL1A* expression (Figure S9G,H,O,P). Thus, these results demonstrate that *THSD7A* activates the *αvβ3/ERK/CEBPD* signaling axis, which transcriptionally regulates *IL1A* to promote endothelial inflammation. In addition, we evaluated the potential role of THSD7A in paracrine signaling, including in macrophages and vascular smooth muscle cells. Single‐cell transcriptomic analysis of human carotid artery plaques revealed that the *αv* subunit (*ITGAV*) of integrin *αvβ3* was upregulated in endothelial cells, macrophages, and smooth muscle cells, while the *β3* subunit showed no significant changes (Figure S10A–D, E–H). Following treatment with rhTHSD7A, there were no significant changes in smooth muscle cell proliferation markers (*MKI67*, *PCNA*), migration markers (*MMP9*, *MMP2*), or apoptosis markers (*BCL2*, *BAX*) (Figure S10I–N). However, rhTHSD7A induced upregulation of the inflammatory cytokines *TNF‐α* and *IL‐1β* in macrophages (Figure S10O,P), suggesting that THSD7A may activate inflammatory signaling in macrophages via paracrine mechanisms.

## 3 Discussion

In this study, we show that *THSD7A* is a positive regulator of endothelial cell inflammation and thereby contributes to the development of atherosclerosis. Mechanistically, *THSD7A* promotes endothelial inflammation and monocyte‐endothelial adhesion via activation of the *αvβ3/CEBPD/IL1A* signaling axis.

THSD7A is recognized as a transmembrane protein with a soluble form, predominantly expressed in renal tissue, spinal neural tissue, placental vascular endothelial cells, and umbilical vein endothelial cells [19, 21, 35]. A previous multicenter GWAS conducted by our team in the Han Chinese population identified a significant association between the SNP rs17165136 in the *THSD7A* gene and CAD; the protective allele of this SNP downregulates *THSD7A* expression [19]. Additionally, a family based genome‐wide linkage scan revealed that *THSD7A* is associated with venous thromboembolism, a disease closely linked to atherosclerotic lesions [25]. Abnormal *THSD7A* expression is also closely correlated with other conditions, including membranous nephropathy, osteoporosis, and tumors [36, 37, 38]. Anti‐THSD7A antibody positivity serves as a clinical marker for evaluating membranous nephropathy. Notably, patients with chronic kidney disease have an increased risk of atherosclerosis related cardiovascular events (e.g., CAD, stroke) [39, 40, 41]. Herein, we demonstrate that the increased *THSD7A* leads to more server atherosclerotic plaques.

Vascular endothelial cells act as a barrier against inflammation. Disruption of endothelial homeostasis leads to the expression of guiding signals (such as adhesion molecules and inflammatory factors), thereby regulating the initiation and progression of atherosclerosis [6, 42, 43, 44]. This is consistent with our observations: in quiescent HUVECs, *THSD7A* knockdown had no effect on inflammation related molecular markers (*ICAM1, SELE, IL6*), whereas *THSD7A* knockdown in activated endothelial cells reduced the expression of these inflammatory markers and attenuated cell adhesion. Single‐cell RNA sequencing of carotid artery plaques revealed that *THSD7A* is predominantly expressed in endothelial cells and its expression is upregulated in endothelial cells from patients’ carotid plaques [45]. Our further bulk RNA sequencing showed that both *THSD7A* overexpression and knockdown are commonly enriched in the “acute inflammatory response” pathway, with *IL1A* being the most significantly altered gene. As a canonical pro‐inflammatory cytokine, *IL1A* promotes atherosclerotic plaque formation by activating endothelial cells and recruiting monocytes [29, 46, 47]. Meanwhile, we noted that *THSD7A* overexpression is mainly enriched in endoplasmic reticulum (ER) stress related pathways; ER dysfunction induces cell apoptosis and inflammation, leading to atherosclerosis [48, 49, 50].

*THSD7A* is highly conserved in humans, mice, and rats. Its RGD (Arg‐Gly‐Asp) motif is considered the binding site for integrin αvβ3 [20, 21]. Previous studies have shown that ligands containing RGD sequences (e.g., cyclic peptides, small molecule mimetics) bind to integrin αvβ3 competitively. This binding inhibits *ERK1/2* signaling and *FAK* (focal adhesion kinase)*/PI3K/Akt* signaling. It thereby reduces endothelial cell survival, migration, and angiogenesis [22, 23, 24]. Furthermore, a soluble form of *THSD7A* promotes endothelial cell migration and angiogenesis via the *FAK, ERK1/2, p38* (p38 mitogen‐activated protein kinase), and *Akt* (Protein Kinase B) signaling pathways. It also regulates cytoskeletal remodeling. This is also thought to be achieved through binding to integrin *αvβ3* [35]. Integrin *αvβ3* has been shown to drive endothelial inflammatory responses and accelerate atherosclerosis progression [51, 52, 53]. The pro‐angiogenic effect of *THSD7A* is consistent with its role in exacerbating atherosclerosis. This is because angiogenesis may promote the progression of atherosclerotic disease and destabilize plaques [27]. However, whether *THSD7A* mediates endothelial inflammation and atherosclerosis in an integrin *αvβ3* dependent manner remains unknown. Our studies demonstrate that THSD7A binds to both the integrin *αv* chain and integrin *β3* chain in endothelial cells. This binding occurs in both resting and activated endothelial cells. In addition, inhibiting integrin *αvβ3* reduces the inflammatory signals promoted by *THSD7A*. Notably, in mice, the integrin *αvβ3* signaling inhibitor Cilengitide reduces *THSD7A* dependent atherosclerosis. These in vivo and in vitro studies collectively demonstrate that integrin *αvβ3* is an essential binding protein. It enables *THSD7A* to promote endothelial inflammation and positively regulate atherosclerosis. These findings also highlight the potential of therapeutic strategies targeting integrin *αvβ3* for atherosclerosis treatment. Of course, our observations were made exclusively in male mice. Given the protective effect of estrogen against atherosclerosis [54, 55, 56], we have not yet included female mice in our studies, and the role of THSD7A in different sexes requires further investigation.

Of particular note, our previous findings showed that *THSD7A* knockdown in monocytes reduced the expression of *ICAM1*, *L‐selectin*, and *ITGB2*. Additionally, we observed positive THSD7A signals in distinct cell types, including macrophages and smooth muscle cells, within mouse atherosclerotic plaques. THSD7A from different cellular sources may participate in the atherosclerotic process through distinct mechanisms. Furthermore, the paracrine role of *THSD7A*, which is predominantly expressed in endothelial cells, cannot be overlooked, given that integrin *αvβ3* is also expressed in smooth muscle cells and macrophages. Although recombinant THSD7A protein had no significant effect on vascular smooth muscle cell proliferation, migration, or apoptosis, it did increase the expression of inflammatory cytokines, including *TNF‐α* and *IL‐1β*, in macrophages. These findings indicate that THSD7A exerts distinct biological functions in autocrine versus paracrine regulatory contexts.

*ERK* is a downstream signaling molecule of integrin *αvβ3*. It plays a crucial regulatory role in cell survival, differentiation, and migration. This role is mainly mediated by the intracellular *FAK/Ras/c‐Raf/ERK* signaling axis [30, 31]. It has been reported that *ERK* can directly regulate the function of nuclear transcription factors *ELK‐1* (ETS domain‐containing protein Elk‐1), *AP‐1* (activator protein 1), *NF‐κB* (Nuclear factor κB). This further mediates the expression of adhesion molecules (*ICAM1*, *VCAM1*, *SELE*) and proinflammatory cytokines *IL6*, *TNF‐α*, *MCP1* (Monocyte chemoattractant protein 1). Thus, *ERK* exerts a core regulatory role in vascular inflammation and atherosclerosis [32, 33, 34]. In our study, overexpression of *THSD7A* increases *ERK* phosphorylation. This phosphorylation is essential for *THSD7A* to exert its functional role. Furthermore, PD98059, an inhibitor of phosphorylated ERK, reduces the *THSD7A* mediated inflammatory effects. These effects include decreased expression of inflammation related molecular markers (*IL1A*, *ICAM1*, *VCAM1*, *SELE*, *IL6*) and reduced cell adhesion. These findings indicate that activation of *ERK* signaling responds to the action of *THSD7A/αvβ3*, thereby exerting a proinflammatory function.

Another key finding of this study is that the transcription factor *CEBPD* is identified as a downstream nodal molecule in the *THSD7A/αvβ3/ERK* pathway. It controls the expression of *IL1A* and the transmission of inflammatory signals. First, overexpression or knockdown of *THSD7A* leads to a corresponding increase or decrease in *CEBPD* expression in endothelial cells. Transcription factor analysis and interference experiments confirm that *IL1A* is a transcriptional target of *CEBPD*. Second, treatment with Cilengitide or PD98059 reduces *THSD7A* promoted expression of *CEBPD* and *IL1A*. It also reduces the expression of inflammation related molecular markers (*ICAM1*, *VCAM1*, *SELE*, *IL6*) and cell adhesion. Finally, inhibition of *CEBPD* reduces *THSD7A* mediated inflammatory signals. These findings strongly support that the *THSD7A/αvβ3/CEBPD/IL1A* signaling axis is novel but crucial for controlling endothelial cell inflammation.

*CEBPD*, a member of the *CEBP* transcription factor family, regulates multiple physiological processes, including inflammation, immune response, oxidative stress, and metabolism [57, 58]. *CEBPD* exhibits higher expression in peripheral blood monocytes of patients with acute myocardial infarction than in those with stable CAD [59]. It has been reported that inflammatory factors like *IL1β* (Interleukin 1 beta) upregulate *CEBPD* expression. This leads to endothelial cell dysfunction [60]. *CEBPD* enhances the expression of *IL1β*, *IL6*, and *CCL2* (C‐C motif chemokine ligand 2). It drives smooth muscle cell phenotypic transition [61]. In macrophages, *p38 MAPK/CREB* (mitogen‐activated protein kinase/cAMP response element‐binding protein) contributes to *CEBPD* activation, thereby promotes lipid accumulation in M1 macrophages [62]. The functional diversity of *CEBPD* depends partly on cell type and cellular environment. Abnormal *CEBPD* expression and activity are closely associated with the development of atherosclerosis and plaque stability [58]. In this study, *CEBPD* regulates endothelial inflammation by controlling the transcription of *IL1A*. This is because *ICAM1*, *VCAM1*, *SELE*, and *IL6* are found to be involved. Specifically, the expression of these molecular markers promotes cell adhesion, which is an early hallmark of atherosclerosis. Interestingly, published studies have shown that after stimulation with the inflammatory factor LPS, *NF‐κB* directly binds to the *CEBPD* promoter [63]. This drives and amplifies the expression of the *IL6* inflammatory gene. These findings confirm the link between *CEBPD* and inflammation. The *THSD7A/αvβ3* driven *IL1A* inflammatory signaling is also regulated by *CEBPD* expression. Therefore, we demonstrate here that *CEBPD* is a hub for *THSD7A/αvβ3/ERK* mediated *IL1A* proinflammatory signaling.

We acknowledge several limitations of the present study. First, we focused the effects of *THSD7A* vascular tissue and found that the decreased *THSD7A* exhibited atheroprotective effects. Such beneficial effect, however, may represent a net effect of multiple organs as the applied mouse model was global knockout of *Thsd7A*. To partially address this issue, we employed an ICAM2 promoter–driven AAV9 strategy to achieve endothelial‐specific *Thsd7a* knockdown; nevertheless, generation of conditional, endothelial‐specific *Thsd7a*‐knockout mice will be required to establish cell‐autonomous contributions definitively. Second, the contribution of *THSD7A* to atherosclerosis in different cell types, as well as whether the mechanisms by which the active forms of *THSD7A* promote atherosclerosis are consistent, remain to be determined. Third, the impact of this signaling axis in human atherosclerosis, particularly in comparison with other known systems, requires further validation to establish its clinical significance, although we have demonstrated that *THSD7A/αvβ3/CEBPD/IL1A* functionally contributed to atherosclerosis and each of those components have been found upregulated significantly in published human carotid plaque datasets [45]. None the less, that the exhibited atheroprotective effects of decreased *THSD7A* is first defined in our study.

In summary, our findings indicate that *THSD7A* exerts pro‐inflammatory effects and exacerbates atherosclerosis. *THSD7A* activated *αvβ3/CEBPD/IL1A* signaling axis represents a novel and critical pathway regulating endothelial inflammation and atherosclerosis. *THSD7A* is not only a genetic marker but also a potential therapeutic target for CAD.

## 4 Experimental Section

### 4.1 Cell Culture

Human monocytic THP‐1 cells (KUNMING CELL BANK. CAS, KCB200549YJ) were maintained in RPMI‐1640 medium (Gibco, cat. 61870036) with 10% fetal bovine serum (FBS; keyGEN, cat. KGL3011‐500) and 1% penicillin/streptomycin (P/S) at 37°C in 5% CO_2_‐humidified air. Cultures were passaged weekly, keeping densities <1 × 107 cells/mL. THP‐1 cells were differentiated into macrophages by treatment with 100 ng/mL phorbol 12‐myristate 13‐acetate (PMA). Human aortic smooth muscle cells (Procell, cat. CP‐H081Y) were maintained in complete DMEM medium. HUVECs (ScienCell, cat. 8000) and HCAECs (Cell Systems, cat. ACBRI 377) were cultured in Endothelial Cell Medium (ECM; ScienCell, cat. 1001) supplemented with 10% FBS, 1% endothelial cell growth supplement, and 1% P/S under the same conditions. These cells were grown on 0.1% gelatin‐precoated flasks, used at passages 3–8, and processed within 48 h of confluence.

### 4.2 Quantitative Real‐Time PCR (qRT‐PCR)

Total RNA from cultured cells or snap‐frozen mouse aortas was isolated using TRIZOL (Bmassay, cat. H0257). One microgram of RNA was reverse‐transcribed into cDNA using a high‐capacity kit (ZOMANBIO, cat. ZR102) per the manufacturer's protocol. Real‐time PCR was performed with SYBR Green 2× Master Mix (Mei5 Biotechnology, cat. MF013‐01) using cycling conditions: 95°C for 5 min (initial denaturation), followed by 40 cycles of 95°C for 10 s and 60°C for 30 s (annealing/extension). Relative expression fold changes were calculated via the 2^‐ΔΔCt method, with *GAPDH* as reference. Primer sequences are shown in Table 2.

**TABLE 2:** List of primers used for qRT‐PCR.

Gene	Forward (from 5' to 3')	Reverse (from 5' to 3')
Human‐*THSD7A*	AAAGAGTGCCAGGTTTCCGA	AACTGCCTGATGGTTCGTGTC
Human‐*IL1A*	AGATGCCTGAGATACCCAAAACC	CCAAGCACACCCAGTAGTCT
Human‐*ICAM1*	CTGACGTGTGCAGTAATACT	GGCTTCGTCAGAATCACGT
Human‐*VCAM1*	GGACCACATCTACGCTGACA	TTGACTGTGATCGGCTTCCC
Human‐*SELE*	GGACACAGCAAATCCCAGTT	CTGCCAGAAGCACTAGGAAG
Human‐*IL6*	CTCCTTCTCCACAAGCGCC	GAAGGCAGCAGGCAACAC
Human‐*αv*	TTCTTCCGATTCCAAACTGG	TGCCTTGCTGAATGAACTTG
Human‐*β3*	TGGCAGCTGTGTCTGTATCC	CCCCGGTCAAACTTCTTACA
Human‐*CEBPD*	CCATGTACGACGACGAGAG	TGTGATTGCTGTTGAAGAGG
Human‐*IL31RA*	CACACTTCGATTCAGGACAGTC	CACATCGCAGAGCTATGACAT
Human‐*GAPDH*	GAGTCAACGGATTTGGTCGT	GACAAGCTTCCCGTTCTCAG
Mouse‐*Thsd7A*	GGGACGTGACGTGCTTAAACA	GGGCTCCATTCCAACCACTC
*IL1A*‐CHIP‐1976∼TSS	TGATATTCTAACCCAAGTTAGCTGT	CCAAAGGTGGTTGTTGAATTTT
*IL1A*‐CHIP‐1741∼TSS	GAAAATGTTATCCCTGTTGAAGC	TATAATGCCTCAGTTCACCAAAG
*IL1A*‐CHIP‐783∼TSS	CTGAGGCCTTACGCATACCTC	TGTAGGATATGCCCAAGGTGTG

### 4.3 SiRNA Transfection and Adenoviral Infection

HUVECs or HCAECs were seeded at 2 × 10^5^cells/well in gelatin‐coated 6 well plates and cultured overnight. At 60%–70% confluence, cells were transfected with 50 nm *THSD7A* targeted or scrambled control siRNA (GenePharma, Suzhou, China) using Lipofectamine 2000 (Invitrogen, Carlsbad, CA, USA) per the manufacturer's protocol. The siRNA sequences are shown in Table 3.

**TABLE 3:** List of siRNA sequences.

Gene	Sense (from 5' to 3')	Antisense (from 5' to 3')
Negative control	UUCUCCGAACGUGUCACGUTT	ACGUGACACGUUCGGAGAATT
si*THSD7A*‐1	CCUCAAAGCCAAUGGACUUTT	AAGUCCAUUGGCUUUGAGGTT
si*THSD7A*‐2	GCCCUAAUGCUGUUGAGAATT	UUCUCAACAGCAUUAGGGCTT
si*IL1A*‐1	CCUUCUAUCAUGUAAGCUATT	UAGCUUACAUGAUAGAAGGTT
si*IL1A*‐2	GGUGCUUAUAAGUCAUCAATT	UUGAUGACUUAUAAGCACCTT
si*CEBPD*‐1	CUCUUCAACAGCAAUCACATT	UGUGAUUGCUGUUGAAGAGTT
si*CEBPD*‐2	CCCUUUGUAUUGUAGAUAATT	UUAUCUACAAUACAAAGGGTT
si*αv*	GCAGGUCUCAGUGUCUCUATT	UAGAGACACUGAGACCUGCTT
si*β3*	GUGUCACCAUACAUGUAUATT	UAUACAUGUAUGGUGACACTT

For overexpression studies, endothelial cells were infected with recombinant adenoviruses encoding THSD7A (Ad‐THSD7A, pAdeno‐MCMV‐THSD7A‐3Flag‐P2A‐EGFP; OBIO, Shanghai, China) or GFP control (Ad‐GFP, pAdeno‐MCMV‐3Flag‐P2A‐EGFP) at the indicated multiplicity of infection (MOI = 10) in endothelial cell medium for 24 h, followed by replacement with complete medium. No drug selection was performed. At 48 h post‐infection, GFP expression was monitored by fluorescence microscopy to confirm transduction efficiency, and THSD7A overexpression was quantified by qPCR and Western blot. For rescue experiments, Ad‐THSD7A infection and si‐IL1A transfection were performed sequentially. Briefly, cells were first infected with Ad‐THSD7A (or Ad‐GFP control); the medium was replaced 48 h post‐infection. Cells were then transfected with si‐IL1A (or control) using Lipofectamine 2000 and harvested 24 h post‐transfection for subsequent analysis. Thus, the total interval from adenoviral infection to cell harvest was 72 h, with 24 h elapsing after si‐IL1A transfection.

### 4.4 Monocyte‐endothelial Cell Adhesion Assay

HUVECs or HCAECs were seeded in 24 well plates and cultured to 80%–90% confluence, then stimulated with TNF‐α (10 ng/mL) or ox‐LDL (100 µg/mL) for 24 h. THP‐1 monocytes (2 × 10^5^ cells/well) were labeled with BCECF‐AM (AAT Bioquest, cat. 21202) for 30 min, then co‐incubated with endothelial cells for 1 h. Non‐adherent monocytes were removed by gentle PBS washing. Monocyte‐endothelial adhesion was visualized via fluorescence microscopy (Carl Zeiss, Oberkochen, Germany). Adherent cells were quantified in 5 replicate fields/well at ×10 magnification using ImageJ software (Image Metrology, Copenhagen, Denmark).

Detailed protocol for an in vitro monocyte‐endothelial cell adhesion assay in mice. After anesthetizing the mice, peripheral blood was collected via cardiac puncture for monocyte isolation. After collecting anticoagulated peripheral blood, it was mixed with an equal volume of RPMI‐1640 complete medium, followed by the addition of mouse monocyte isolation solution (Solarbio, cat. P5230), and a monocyte layer was isolated using Ficoll‐Paque density gradient centrifugation (horizontal centrifuge, 20°C, 800g, 30 min). Monocytes were harvested from this layer and subsequently washed twice with PBS, centrifuging at 600g for 5 min each time. The isolated monocytes were cultured in RPMI‐1640 medium containing 10% fetal bovine serum and 1% penicillin/streptomycin (P/S) in a cell culture incubator at 37°C and 5% CO_2_. The monocytes required no induction or differentiation and were cultured and passaged under standard conditions for use in cell adhesion assays. Following blood collection, the mice were perfused transcardially with PBS to remove residual circulating blood. The aorta was isolated, and adipose and connective tissues were rapidly stripped under a dissecting microscope. The aorta was opened longitudinally and cut into 0.5‐cm segments. After being moistened with complete endothelial cell medium (ECM), the segments were placed endothelial‐side down in a dry 3.5‐cm culture dish. After standing for 1 h to allow the vessel segments to adhere firmly to the dish, complete ECM was added. The tissues were cultured for approximately 4–5 days to allow endothelial cells to migrate out, attach, and fully spread. The aortic segments were then carefully removed, and the cells were cultured for an additional 2 days until they reached contact inhibition. The cells were then harvested and seeded into 96‐well plates for the adhesion assay. Notably, for *Thsd7A*‐knockout mice, the endothelial cells were cultured for 48 h until a uniform monolayer was formed before the adhesion assay. For the overexpression experiment, we re‐transfected the cells with Ad‐THSD7A to ensure overexpression and performed the monocyte adhesion assay 48 h after transfection. Mouse monocytes (1 × 10^6^ cells/mL) separated by density gradient centrifugation and labeled with BCECF‐AM were added to a monolayer of mouse aortic endothelial cells (100 µL of cell suspension per well in a 96‐well plate) and incubated for 1 h at 37°C in a 5% CO_2_ environment. Wash three times with PBS and gently remove unadhered cells. After fixation, observe the adherent monocytes under a fluorescence microscope and randomly select five fields of view from each well for counting to facilitate statistical analysis.

BCECF‐AM (2′,7′‐bis‐(2‐carboxyethyl)‐5‐(and‐6)‐carboxyfluorescein acetoxymethyl ester), although pH‐sensitive, is a cell‐permeant fluorescent probe that is hydrolyzed by intracellular esterases to membrane‐impermeant BCECF, thereby becoming trapped within viable cells. The use of BCECF‐AM in the present adhesion assay was based on the following considerations: (1) its high fluorescence intensity and excellent photostability, which facilitate fluorescence microscopic visualization and quantification; (2) its low cytotoxicity, which does not impair monocyte adhesion capacity; and (3) its uniform fluorescence distribution after labelling, enabling clear discrimination from background.

### 4.5 Protein Extraction and Western Blotting

Cells and tissues were lysed on ice in RIPA buffer with 1× complete protease inhibitor cocktail (Roche, cat. 4693159001) and 1× phosphatase inhibitor (Beyotime, cat. P1081). Protein concentration was measured via BCA assay (Thermo Fisher Scientific, cat. A55860). Equal protein amounts (20–40µg) were resolved on 8% SDS‐PAGE gels and electrotransferred to 0.45 µm PVDF membranes. Membranes were blocked for 1 h at room temperature in 5% skim milk/TBST, then incubated overnight at 4°C with primary antibodies: THSD7A (Bioss, cat. bs8749R, 1:500; customized), phospho‐ERK (CST, cat. 5726, 1:1000), total ERK (Santa Cruz, cat. sc‐81459, 1:1000), αv (Abcam, cat. ab179475, 1:1000), β3 (Santa Cruz, cat. sc‐365546, 1:1000), Flag (Proteintech, cat. 66008‐4‐Ig, 1:1000), and GAPDH (Proteintech, cat. 1E6D9, 1:5000). After three washes with TBST, membranes were incubated with HRP‐conjugated secondary antibodies (Proteintech, cat. SA00001‐1, SA00001‐2, 1:5000) for 1 h at room temperature. Protein bands were visualized with ECL substrate (Thermo Fisher Scientific, cat. 32106) to assess expression levels.

### 4.6 Immunofluorescence Staining

Cells or en face aortic preparations were fixed in 4% paraformaldehyde for 15 min at RT, and permeabilized with 0.1% Triton X‐100 in PBS for 10 min. Non‐specific binding was blocked with 10% serum in PBS for 1 h at RT. Samples were then incubated overnight at 4°C with primary antibodies: anti‐THSD7A, anti‐CD31 (Thermo, cat. AF3628). The next day, samples were incubated with fluorescent secondary antibodies for 1 h at RT, followed by counterstaining with Hoechst 33342 (Abcam, cat. ab104139) for 30 min. Images were acquired via a Zeiss LSM 880 confocal microscope with identical settings across all conditions.

### 4.7 Co‐Immunoprecipitation (Co‐IP) Assay

Endothelial cells in quiescent state or those transduced with Ad‐THSD7A were lysed after 48 h using IP buffer supplemented with fresh protease inhibitor mixture. Lysates were incubated overnight at 4°C with control IgG, Flag, αv and β3 antibody to form protein‐antibody complexes, then immunoprecipitated using protein A/G agarose beads (Santa Cruz, cat. sc‐2003). Immunoprecipitates were washed with IP buffer, and proteins resolved by SDS‐PAGE, followed by immunoblotting (IB) with appropriate primary antibodies.

### 4.8 Chromatin Immunoprecipitation (ChIP)

Collected endothelial cells (HUVECs or HCAECs) were cross‐linked with 1% formaldehyde (Sigma–Aldrich, cat. F8775) at room temperature for 15 min, followed by quenching the cross‐links with 2.5 mol/L glycine at room temperature for 5 min. Following cell lysis, chromatin was sonicated for 6 min to generate 150–500 bp fragments. Chromatin DNA was prepared using a commercial kit (Cell Signaling Technology, cat. 9003S). Immunoprecipitated protein‐DNA complexes were isolated from chromatin using the CEBPD monoclonal antibody (Santa Cruz, cat. sc‐365546) or control mouse IgG (Santa Cruz, cat. sc‐515946). PCR amplification of reverse crosslinked immunoprecipitated chromatin was performed using primers detailed in Table 1.

### 4.9 Luciferase Reporter Assay

Sequences containing the *CEBPD* gene (NM_005195.4), *CEBPD* binding sequence of *IL1A* gene (NM_000575.5), or mutant *IL1A* promoter sequences were subcloned into the pGL6‐basic luciferase reporter vector. All constructs were verified by DNA sequencing. Luciferase activity was measured 48 h after transfection of HEK293T cells using the Dual‐Luciferase Reporter Assay System (Promega, cat. N1610) and normalized to Renilla luciferase activity.

### 4.10 RNA‐Sequencing Analysis

Following 48 h of *THSD7A* overexpression or knockdown in HUVECs, cells were harvested and total RNA was extracted. RNA sequencing was performed by the Beijing Genomics Institute (BGI, Beijing, China). Fastq data were aligned to the human reference genome using Bowtie2 (v2.25). Gene expression levels were quantified using RSEM (v1.2.12). Differential expression analysis was conducted with DESeq2 (v1.4.5), using thresholds of Log2 fold change ≥ 1 and False Discovery Rate (FDR) < 0.05. Heatmaps of gene expression profiles were generated using pheatmap (v1.0.12). Gene Ontology (GO) and Kyoto Encyclopedia of Genes and Genomes (KEGG) enrichment analyses were performed using the web‐based GEne SeT AnaLysis Toolkit (GESTALT).

### 4.11 Single Cell‐Type Expression Analysis

Single‐cell RNA sequencing data were obtained from the Gene Expression Omnibus database (Dataset ID: GSE159677). Using the Seurat package, we standardized the single‐cell sequencing data to obtain accurate and unbiased single‐cell gene expression data. Subsequently, we applied PCA (Principal Component Analysis) and UMAP (Uniform Manifold Approximation and Projection) algorithms for dimensionality reduction. Cell type annotation was performed based on canonical marker genes identified by COSG algorithm.

### 4.12 Animals

All animal procedures were conducted in accordance with the NIH Guide for the Care and Use of Laboratory Animals (8th edition), and approved by the Institutional Animal Care and Use Committee of Nanchang University (protocol no: NCULAE‐20221130017). Global *Thsd7A^−/−^* mice were generated on the C57BL/6J wild‐type (WT) genetic background. Following six generations of backcrossing, littermate mice were randomly assigned to subsequent experiments. 8‐week‐old mice (22–25 g) were housed in individually ventilated cages under specific pathogen free (SPF) conditions (22°C ± 1°C, 45%–55% humidity), with a 12 h light/12 h dark cycle and ad libitum access to autoclaved chow and water.

All male mice were anesthetized once with an intraperitoneal injection of sodium pentobarbital at a dose of 50 mg/kg body weight. For euthanasia, mice were humanely euthanized by controlled exposure to medical‐grade carbon dioxide (30% chamber volume/min) in a sealed chamber. Gradual displacement to ≥70% CO_2_ concentration induced rapid deep anesthesia and death with minimal distress. Following respiratory arrest, flow was maintained for ≥1 min to ensure irreversible euthanasia.

### 4.13 Serum Biochemical Analysis

Serum samples were obtained from mice by centrifuging whole blood at 3000 rpm for 15 min at 4°C, following clotting at room temperature for 30 min. The lipid profile, including total cholesterol (TC), triglycerides (TG), high‐density lipoprotein cholesterol (HDL‐C), and low‐density lipoprotein cholesterol (LDL‐C), was quantified using commercially available assay kits (cat. A111‐1‐1, A113‐2‐1, A112‐1‐1, A1101‐1; Jiancheng, Nanjing, China) in accordance with the manufacturer's instructions.

### 4.14 Overexpression and Knockdown of THSD7A in Mice

Adenoviral vectors encoding full‐length human *THSD7A* under CMV promoter control were constructed. The expression cassette (pAdeno‐MCMV‐THSD7A‐3×Flag‐P2A‐EGFP) and transgene‐lacking control vector (pAdeno‐MCMV‐3×Flag‐P2A‐EGFP) were packaged by OBIO Technology (Shanghai, China). Viral particles were quantified via qPCR for genomic content and adjusted to 1.0×10^1^ ^1^ PFU/mL in sterile saline. Eight‐week‐old male *ApoE^−/−^* mice received 200 µL of respective vectors via lateral tail vein every 14 days (3 total injections). After 6 weeks, aortas and hearts were excised for analyses. For endothelial‐specific knockdown, Eight‐week‐old male *ApoE^−/−^* mice were treated with an ICAM‐2 promoter‐driven, endothelial cell‐specific AAV9 vector (AAV9‐EC‐sh*Thsd7A*; 1×10^1^ ^2^ vg per mouse; Shandong Weizhen Biotechnology) to achieve endothelial cell‐specific knockdown of *Thsd7A*. The extent of atherosclerotic lesions was assessed 6 weeks later.

### 4.15 Drug Treatments

Cilengitide (MCE, cat. HY‐16141), a selective αvβ3 integrin antagonist, was dissolved in sterile saline and injected into mice via tail vein at 10 µg/g once weekly for 6 weeks. After treatment, mice were anesthetized, euthanized by exsanguination, and aortae/hearts harvested en bloc for en face Oil Red O staining and atherosclerotic lesion histology.

For in vitro studies, cells were treated with cilengitide at a final concentration of 1 µm to assess inflammatory changes. Additionally, cells were treated with an ERK inhibitor (PD98059, MCE, cat. HY‐12028) and an *IL1A* receptor antagonist (IL1RA, MCE, cat. HY‐P7029) at final concentrations of 10 µm and 10 ng/mL, respectively.

### 4.16 Atherosclerotic Lesion Analysis

All mice were fed a high‐fat diet (HFD; Per 4,056 kcal: 40 kcal% fat, 11.25 g cholesterol, 45 g carbohydrates, and 23 g protein. SYSE BIO, cat. D12108C) for 6 weeks prior to the experiment. The hearts and aortas from anesthetized mice were fixed in 4% paraformaldehyde at 4°C overnight. After fixation, adventitial fat and connective tissue were dissected from aortas under a dissecting microscope. Aortas were longitudinally incised to expose the lumen (including subclavian, right/left common carotid arteries) and stained with 0.2% Oil Red O (Sigma–Aldrich, cat. O9755). Hearts with aortic roots were embedded in optimal cutting temperature (OCT) compound. Serial 5‐µm aortic root sections were prepared and Oil Red O stained per standard protocols. Lesion areas were quantified via Image software.

### 4.17 Statistical Analysis

Data are expressed as mean ± standard error of the mean (SEM). The Shapiro‐Wilk test was used to assess normality of data distribution. For comparisons between two groups, statistical significance was evaluated using a two‐tailed unpaired Student's *t*‐test (for normally distributed data) or Mann–Whitney test (for non‐normally distributed data). For single‐factor experiments with more than two groups, one‐way analysis of variance (ANOVA) was applied followed by Tukey's post‐hoc test. Two‐way ANOVA with Tukey's multiple‐comparisons correction was used for analyses involving two or more factors. All statistical analyses were performed using GraphPad Prism 8 software (GraphPad Software, San Diego, CA, USA). *p*‐value < 0.05 was considered statistically significant. Each data point in the figures represents an independent biological replicate.

## Author Contributions

Conceptualization and Supervision, X.L.T.; Experiments: J.K.L., H.F.L., Y.Q.W. A.D.W. J.Y.Q., X.T.G., Y.Z., Y.Y.L., Y.Y.Z., B.B.Z., F.W.; Writing – Original Draft Preparation, J.K.L., H.F.L., Y.Z.Z., W.X.L., X.E.L, Q.Y.; Writing – Review and Editing, X.L.T. All authors have read and agreed to the published version of the manuscript.

## Funding

This study was funded by the Key Program of the National Natural Science Foundation of China (82330046) and the Jiangxi Province Key Laboratory of Aging and Disease (2024SSY07161).

## Conflicts of Interest

The authors declare no conflicts of interest.

## Supporting information




**Supporting File**: advs77422‐sup‐0001‐SuppMat.docx.

## Data Availability

The datasets generated and/or analyzed during the current study are available from the corresponding author upon reasonable request.

## References

[1] P. Libby , J. E. Buring , L. Badimon , et al., “Atherosclerosis,” Nature Reviews Disease Primers 5 (2019): 56. 10.1038/s41572-019-0106-z 31420554

[2] G. A. Mensah , V. Fuster , C. J. L. Murray , et al., “Global Burden of Cardiovascular Diseases and Risks, 1990‐2022,” Journal of the American College of Cardiology 82, no. 25 (2023): 2350–2473. 10.1016/j.jacc.2023.11.007 PMC7615984 38092509

[3] X. Zhou , L. Yu , Y. Zhao , and J. Ge , “Panvascular Medicine: an Emerging Discipline Focusing on Atherosclerotic Diseases,” European Heart Journal 43, no. 43 (2022): 4528–4531. 10.1093/eurheartj/ehac448 35947920

[4] R. Ross , “The Pathogenesis of Atherosclerosis: a Perspective for the 1990s,” Nature 362, no. 6423 (1993): 801–809. 10.1038/362801a0 8479518

[5] J. Davignon and P. Ganz , “Role of Endothelial Dysfunction in Atherosclerosis,” Circulation 109, no. 23 (2004): 27–32. 10.1161/01.CIR.0000131515.03336.f8 15198963

[6] M. A. Gimbrone and G. García‐Cardeña , “Endothelial Cell Dysfunction and the Pathobiology of Atherosclerosis,” Circulation Research 118, no. 4 (2016): 620–636. 10.1161/CIRCRESAHA.115.306301 PMC4762052 26892962

[7] S. Xu , I. Ilyas , P. J. Little , et al., “Endothelial Dysfunction in Atherosclerotic Cardiovascular Diseases and beyond: from Mechanism to Pharmacotherapies,” Pharmacological Reviews 73, no. 3 (2021): 924–967. 10.1124/pharmrev.120.000096 34088867

[8] A. Abbate , S. Toldo , C. Marchetti , J. Kron , B. W. Van Tassell , and C. A. Dinarello , “Interleukin‐1 and the Inflammasome as Therapeutic Targets in Cardiovascular Disease,” Circulation Research 126, no. 9 (2020): 1260–1280. 10.1161/CIRCRESAHA.120.315937 PMC8760628 32324502

[9] S. C. Bergheanu , M. C. Bodde , and J. W. Jukema , “Pathophysiology and Treatment of Atherosclerosis,” Netherlands Heart Journal 25, no. 4 (2017): 231–242. 10.1007/s12471-017-0959-2 PMC5355390 28194698

[10] M. Fischer , U. Broeckel , S. Holmer , et al., “Distinct Heritable Patterns of Angiographic Coronary Artery Disease in Families with Myocardial Infarction,” Circulation 111, no. 7 (2005): 855–862. 10.1161/01.CIR.0000155611.41961.BB 15710764

[11] P. F. Martinez and M. P. Okoshi , “Genetic Risk in Coronary Artery Disease,” Arquivos Brasileiros De Cardiologia 111, no. 1 (2018): 62–63. 10.5935/abc.20180130 PMC6078374 30110045

[12] D. Vaidya , L. R. Yanek , T. F. Moy , T. A. Pearson , L. C. Becker , and D. M. Becker , “Incidence of Coronary Artery Disease in Siblings of Patients with Premature Coronary Artery Disease: 10 Years of Follow‐up,” The American Journal of Cardiology 100, no. 9 (2007): 1410–1415. 10.1016/j.amjcard.2007.06.031 PMC2128738 17950799

[13] S. Zdravkovic , A. Wienke , N. L. Pedersen , M. E. Marenberg , A. I. Yashin , and U. De Faire , “Heritability of Death from Coronary Heart Disease: a 36‐Year Follow‐up of 20 966 Swedish Twins,” Journal of Internal Medicine 252, no. 3 (2002): 247–254. 10.1046/j.1365-2796.2002.01029.x 12270005

[14] S. M. Rappaport and A. M. Neophytou , “Genetic Risk, Lifestyle, and Coronary Artery Disease,” New Engl J Med 376, no. 12 (2017): 1193–1194. 10.1056/NEJMc1700362 28328342

[15] Y. Li , H. Yan , J. Guo , et al., “Down‐regulated RGS5 by Genetic Variants Impairs Endothelial Cell Function and Contributes to Coronary Artery Disease,” Cardiovascular Research 117, no. 1 (2021): 240–255. 10.1093/cvr/cvz268 31605122

[16] S. R. Archacki , G. Angheloiu , X.‐L. Tian , et al., “Identification of New Genes Differentially Expressed in Coronary Artery Disease by Expression Profiling,” American Journal of Human Genetics 73, no. 5 (2003): 165–165. 10.1152/physiolgenomics.00181.2002 12902549

[17] F. Jiang , Y. Dong , C. Wu , et al., “Fine Mapping of Chromosome 3q22.3 Identifies Two Haplotype Blocks in ESYT3 Associated with Coronary Artery Disease in Female Han Chinese,” Atherosclerosis 218, no. 2 (2011): 397–403. 10.1016/j.atherosclerosis.2011.06.017 21733517

[18] J.‐F. Ma , F. Yang , S. N. Mahida , et al., “TBX5 mutations Contribute to Early‐onset Atrial Fibrillation in Chinese and Caucasians,” Cardiovascular Research 109, no. 3 (2016): 442–450. 10.1093/cvr/cvw003 PMC4752043 26762269

[19] Y. Li , D. W. Wang , Y. D. Chen , et al., “Genome‐Wide Association and Functional Studies Identify SCML4 and THSD7A as Novel Susceptibility Genes for Coronary Artery Disease,” Arterioscl Throm Vas 38, no. 4 (2018): 964–975. 10.1161/ATVBAHA.117.310594 29472232

[20] M. Fresquet , S. J. Rhoden , T. A. Jowitt , et al., “Autoantigens PLA2R and THSD7A in Membranous Nephropathy Share a Common Epitope Motif in the N‐terminal Domain,” Journal of Autoimmunity 106 (2020): 102308. 10.1016/j.jaut.2019.102308 PMC7471840 31395435

[21] C.‐H. Wang , P.‐T. Su , X.‐Y. Du , et al., “Thrombospondin Type I Domain Containing 7A (THSD7A) Mediates Endothelial Cell Migration and Tube Formation,” Journal of Cellular Physiology 222, no. 3 (2010): 685–694. 10.1002/jcp.21990 20020485

[22] S. Maubant , D. Saint‐Dizier , M. Boutillon , et al., “Blockade of αvβ3 and αvβ5 Integrins by RGD Mimetics Induces Anoikis and Not Integrin‐mediated Death in human Endothelial Cells,” Blood 108, no. 9 (2006): 3035–3044. 10.1182/blood-2006-05-023580 16835373

[23] E. Lambert , E. Fuselier , L. Ramont , B. Brassart , S. Dukic , and J.‐B. Oudart , “Conformation‐dependent Binding of a Tetrastatin Peptide to αvβ3 Integrin Decreases Melanoma Progression through FAK/PIK/Akt Pathway Inhibition,” Sci Rep‐Uk 8 (2018): 9837. 10.1038/s41598-018-28003-x PMC6026150 29959360

[24] T. M. Danilucci , P. K. Santos , B. C. Pachane , G. F. D. Pisani , R. L. B. Lino , and B. C. Casali , “Recombinant RGD‐disintegrin DisBa‐01 Blocks Integrin αvβ3 and Impairs VEGF Signaling in Endothelial Cells,” Cell Communication and Signaling 17 (2019): 27. 10.1186/s12964-019-0339-1 PMC6425665 30894182

[25] H. de Visser M C , R. van Minkelen , V. van Marion , et al., “Genome‐wide Linkage Scan in Affected Sibling Pairs Identifies Novel Susceptibility Region for Venous Thromboembolism: Genetics in Familial Thrombosis Study,” Journal of Thrombosis and Haemostasis 11, no. 8 (2013): 1474–1484. 10.1111/jth.12313 23742623

[26] J. Rayes and A. Brill , “Hot under the Clot: Venous Thrombogenesis Is an Inflammatory Process,” Blood 144, no. 5 (2024): 477–489. 10.1182/blood.2023022522 38728383

[27] C. H. Chen and J. P. Walterscheid , “Plaque Angiogenesis versus Compensatory Arteriogenesis in Atherosclerosis,” Circulation Research 99, no. 8 (2006): 787–789. 10.1161/01.RES.0000247758.34085.a6 17038645

[28] A. D. Mahmoud , M. D. Ballantyne , V. Miscianinov , et al., “The Human‐Specific and Smooth Muscle Cell‐Enriched LncRNA SMILR Promotes Proliferation by Regulating Mitotic CENPF mRNA and Drives Cell‐Cycle Progression Which Can Be Targeted to Limit Vascular Remodeling,” Circulation Research 125, no. 5 (2019): 535–551. 10.1161/CIRCRESAHA.119.314876 PMC6693924 31339449

[29] S. J. Schunk , S. Triem , D. Schmit , et al., “Interleukin‐1α Is a Central Regulator of Leukocyte‐Endothelial Adhesion in Myocardial Infarction and in Chronic Kidney Disease,” Circulation 144, no. 11 (2021): 893–908. 10.1161/CIRCULATIONAHA.121.053547 34192892

[30] J. D. Hood , R. Frausto , W. B. Kiosses , M. A. Schwartz , and D. A. Cheresh , “Differential Αv Integrin–mediated Ras‐ERK Signaling during Two Pathways of Angiogenesis,” The Journal of Cell Biology 162, no. 5 (2003): 933–943. 10.1083/jcb.200304105 PMC2172815 12952943

[31] S. K. Mitra , D. A. Hanson , and D. D. Schlaepfer , “Focal Adhesion Kinase: in Command and Control of Cell Motility,” Nature Reviews Molecular Cell Biology 6, no. 1 (2005): 56–68. 10.1038/nrm1549 15688067

[32] S. Dragoni , N. Hudson , B.‐A. Kenny , et al., “Endothelial MAPKs Direct ICAM‐1 Signaling to Divergent Inflammatory Functions,” The Journal of Immunology 198, no. 10 (2017): 4074–4085. 10.4049/jimmunol.1600823 PMC5421301 28373581

[33] M. Peng , R. Sun , Y. Hong , et al., “Extracellular Vesicles Carrying Proinflammatory Factors May Spread Atherosclerosis to Remote Locations,” Cellular and Molecular Life Sciences 79, no. 8 (2022): 430. 10.1007/s00018-022-04464-2 PMC11071964 35851433

[34] Q. Li , L. Yu , A. Gao , et al., “METTL3 (Methyltransferase Like 3)‐Dependent N6‐Methyladenosine Modification on Braf mRNA Promotes Macrophage Inflammatory Response and Atherosclerosis in Mice,” Arteriosclerosis, Thrombosis, and Vascular Biology 43, no. 5 (2023): 755–773. 10.1161/ATVBAHA.122.318451 36951060

[35] M.‐W. Kuo , C.‐H. Wang , H.‐C. Wu , S.‐J. Chang , and Y.‐J. Chuang , “Soluble THSD7A Is an N‐Glycoprotein That Promotes Endothelial Cell Migration and Tube Formation in Angiogenesis,” PLoS ONE 6, no. 12 (2011): 29000. 10.1371/journal.pone.0029000 PMC3237571 22194972

[36] S. Mori , I. Kou , H. Sato , et al., “Association of Genetic Variations of Genes Encoding Thrombospondin, Type 1, Domain‐containing 4 and 7A with Low Bone Mineral Density in Japanese Women with Osteoporosis,” Journal of Human Genetics 53, no. 8 (2008): 694–697. 10.1007/s10038-008-0300-4 18488137

[37] N. M. Tomas , L. H. Beck , C. Meyer‐Schwesinger , et al., “Thrombospondin Type‐1 Domain‐Containing 7A in Idiopathic Membranous Nephropathy,” New England Journal of Medicine 371, no. 24 (2014): 2277–2287. 10.1056/NEJMoa1409354 PMC4278759 25394321

[38] P. R. Stahl , E. Hoxha , T. Wiech , C. Schröder , R. Simon , and R. A. K. Stahl , “THSD7A expression in human Cancer,” Genes, Chromosomes and Cancer 56, no. 4 (2017): 314–327. 10.1002/gcc.22440 28035718

[39] P. Kouis , A. Kousios , A. Kanari , D. Kleopa , S. I. Papatheodorou , and A. G. Panayiotou , “Association of Non‐invasive Measures of Subclinical Atherosclerosis and Arterial Stiffness with Mortality and Major Cardiovascular Events in Chronic Kidney Disease: Systematic Review and Meta‐analysis of Cohort Studies,” Clinical Kidney Journal 13, no. 5 (2020): 842–854. 10.1093/ckj/sfz095 PMC7849940 33542824

[40] E. Di Angelantonio , R. Chowdhury , N. Sarwar , T. Aspelund , J. Danesh , and V. Gudnason , “Chronic Kidney Disease and Risk of Major Cardiovascular Disease and Non‐vascular Mortality: Prospective Population Based Cohort Study,” Bmj‐Brit Med J 341 (2010): c4986. 10.1136/bmj.c4986 PMC2948649 20884698

[41] A. Attar and M. Sayadi , “Effect of Chronic Kidney Disease on Cardiovascular Events, an Epidemiological Aspect from SPRINT Trial,” Iran J Kidney Dis 13, no. 5 (2019): 329–337. 31705750

[42] R. Ross , “Atherosclerosis ‐ An Inflammatory Disease ‐ Reply,” New Engl J Med 340, no. 24 (1999): 1929–1929. 10375320

[43] M. Jia , Q. Li , J. Guo , et al., “Deletion of BACH1 Attenuates Atherosclerosis by Reducing Endothelial Inflammation,” Circulation Research 130, no. 7 (2022): 1038–1055. 10.1161/CIRCRESAHA.121.319540 35196865

[44] S. Ding , J. Liu , X. Han , et al., “ICAM‐1‐related Noncoding RNA Accelerates Atherosclerosis by Amplifying NF‐κB Signaling,” Journal of Molecular and Cellular Cardiology 170 (2022): 75–86. 10.1016/j.yjmcc.2022.06.001 35714558

[45] T. Alsaigh , D. Evans , D. Frankel , and A. Torkamani , “Decoding the Transcriptome of Calcified Atherosclerotic Plaque at Single‐cell Resolution,” Communications Biology 5, no. 1 (2022): 1084. 10.1038/s42003-022-04056-7 PMC9556750 36224302

[46] L. F. Buckley and A. Abbate , “Interleukin‐1 Blockade in Cardiovascular Diseases: a Clinical Update,” European Heart Journal 39, no. 22 (2018): 2063–2069. 10.1093/eurheartj/ehy128 29584915

[47] C. Maeder , T. Speer , A. Wirth , et al., “Membrane‐bound Interleukin‐1α Mediates Leukocyte Adhesion during Atherogenesis,” Frontiers in Immunology 14 (2023): 1252384. 10.3389/fimmu.2023.1252384 PMC10494239 37701434

[48] G. S. Hotamisligil , “Endoplasmic Reticulum Stress and Atherosclerosis,” Nature Medicine 16, no. 4 (2010): 396–399. 10.1038/nm0410-396 PMC2897068 20376052

[49] S. J. Yang , M. Wu , X. Y. Li , et al., “Role of Endoplasmic Reticulum Stress in Atherosclerosis and Its Potential as a Therapeutic Target,” Oxid Med Cell Longev 2020 (2020): 9270107. 10.1155/2020/9270107 PMC7499294 32963706

[50] J. Ren , Y. Bi , J. R. Sowers , C. Hetz , and Y. Zhang , “Endoplasmic Reticulum Stress and Unfolded Protein Response in Cardiovascular Diseases,” Nature Reviews Cardiology 18, no. 7 (2021): 499–521. 10.1038/s41569-021-00511-w 33619348

[51] F. Fang , T. Feng , J. Li , et al., “Cathepsin K Contributed to Disturbed Flow‐induced Atherosclerosis Is Dependent on Integrin‐actin Cytoskeleton–NF–κB Pathway,” Genes & Diseases 10, no. 2 (2023): 583–595. 10.1016/j.gendis.2022.03.020 PMC10201601 37223522

[52] W. S. Jenkins , A. T. Vesey , A. Vickers , et al., “In Vivo Alpha‐V Beta‐3 Integrin Expression in human Aortic Atherosclerosis,” Heart 105, no. 24 (2019): 1868–1875. 10.1136/heartjnl-2019-315103 PMC6929706 31422361

[53] J. Chen , J. Green , A. Yurdagul , P. Albert , M. C. McInnis , and A. W. Orr , “αvβ3 Integrins Mediate Flow‐Induced NF‐κB Activation, Proinflammatory Gene Expression, and Early Atherogenic Inflammation,” The American Journal of Pathology 185, no. 9 (2015): 2575–2589. 10.1016/j.ajpath.2015.05.013 PMC4597278 26212910

[54] J. B. Hodgin , J. H. Krege , R. L. Reddick , K. S. Korach , O. Smithies , and N. Maeda , “Estrogen Receptor α Is a Major Mediator of 17β‐estradiol's Atheroprotective Effects on Lesion Size in Apoe–/– mice,” Journal of Clinical Investigation 107, no. 3 (2001): 333–340. 10.1172/JCI11320 PMC199197 11160157

[55] M. R. Meyer , N. C. Fredette , T. A. Howard , et al., “G Protein‐coupled Estrogen Receptor Protects from Atherosclerosis,” Scientific Reports 4, no. 1 (2014): 7564. 10.1038/srep07564 PMC4274506 25532911

[56] A. C. Villablanca , A. Tenwolde , M. Lee , M. Huck , S. Mumenthaler , and J. C. Rutledge , “17β‐Estradiol Prevents Early‐Stage Atherosclerosis in Estrogen Receptor‐Alpha Deficient Female Mice,” Journal of Cardiovascular Translational Research 2, no. 3 (2009): 289–299. 10.1007/s12265-009-9103-z PMC2719738 19654889

[57] L. Wang , J. Feng , Y. Deng , et al., “CCAAT/Enhancer‐Binding Proteins in Fibrosis: Complex Roles beyond Conventional Understanding,” Research 2022 (2022): 9891689. 10.34133/2022/9891689 PMC9575473 36299447

[58] T. J. Li , S. L. Lin , Y. Y. Zhu , D. Ye , X. Rong , and L. Wang , “Basic Biology and Roles of CEBPD in Cardiovascular Disease,” Cell Death Discovery 11, no. 1 (2025): 102. 10.1038/s41420-025-02357-4 PMC11909146 40087290

[59] X. Tang , Y. Zhou , Z. Chen , et al., “Identification of Key Biomarkers for Predicting CAD Progression in Inflammatory Bowel Disease via Machine‐Learning and Bioinformatics Strategies,” Journal of Cellular and Molecular Medicine 28, no. 6 (2024): 18175. 10.1111/jcmm.18175 PMC10919158 38451044

[60] N. T. Hogan , M. B. Whalen , L. K. Stolze , et al., “Transcriptional Networks Specifying Homeostatic and Inflammatory Programs of Gene Expression in human Aortic Endothelial Cells,” Elife 6 (2017): 22536. 10.7554/eLife.22536 PMC5461113 28585919

[61] Q. Wang , H. G. Ozer , B. Wang , et al., “A Hierarchical and Collaborative BRD4/CEBPD Partnership Governs Vascular Smooth Muscle Cell Inflammation,” Molecular Therapy ‐ Methods & Clinical Development 21 (2021): 54–66. 10.1016/j.omtm.2021.02.021 PMC7966960 33768129

[62] H.‐Y. Lai , L.‐W. Hsu , H.‐H. Tsai , et al., “CCAAT/Enhancer‐binding Protein Delta Promotes Intracellular Lipid Accumulation in M1 Macrophages of Vascular Lesions,” Cardiovascular Research 113, no. 11 (2017): 1376–1388. 10.1093/cvr/cvx134 28859294

[63] V. Litvak , S. A. Ramsey , A. G. Rust , et al., “Function of C/EBPδ in a Regulatory Circuit That Discriminates between Transient and Persistent TLR4‐induced Signals,” Nature Immunology 10, no. 4 (2009): 437–443. 10.1038/ni.1721 PMC2780024 19270711

