# Supplementary material for: THSD7A Exacerbates Atherosclerosis via Activation of Signaling Axis αvβ3/CEBPD/IL1A
Source: Adv Sci (Weinh). 2026 Aug 24:e77422. Online ahead of print. doi: 10.1002/advs.77422 (PMC13502345; doi:10.1002/advs.77422)
Supplement: Supplementary file 1 [file ADVS-9999-e77422-s001.docx]

**Supporting Information**

***THSD7A* Exacerbates Atherosclerosis via Activation of Signaling Axis *αvβ3/CEBPD/IL1A***

*Jiankun Liu, Hongfei Liu, Yiqi Wan, Andong Wu, Jiayu Qiu, Xueting Gong, Ya Zhao, Yuanyuan Li, Bingbing Zhou, Yangyi Zheng, Fang Wu, Yuanzheng Zhu, Weixin Lv, Xueer Li, Qiang Yuan, Xiao-Li Tian^*^*

J.K.L. Author 1, Y.Q.W. Author 3, A.D.W.Author 4, J.Y.Q.Author 5, X.T.G. Author 6, Y.Z. Author 7, Y.Y.L. Author 8, B.B.Z. Author 9, Y.Y.Z. Author 10, F.W. Author 11, Y.Z.Z. Author 12, W.X.L. Author 13, X.E.L. Author 14, Q.Y. Author 15, X.L.T. Corresponding author.

Aging and Vascular Diseases, Human Aging Research Institute and School of Life Science, Nanchang University, and Jiangxi Key Laboratory of Aging and Diseases, Nanchang, China

H.F.L. Author 2

Vascular Function Laboratory, Human Aging Research Institute and School of Life Science, Nanchang university, and Jiangxi Key Laboratory of Human Aging, Nanchang, China

*Corresponding to: X.L.T., Email: tianxiaoli@ncu.edu.cn

Address: Human Aging Research Institute and School of Life Science, Nanchang University, 999 Xuefu Street, Honggutan New District, Nanchang city, Jiangxi Province 330031, China

**Figure S1.** Localization and expression of THSD7A in blood vessels detected by in vivo confocal microscopy. (A) Tracking and localization of green fluorescent protein GFP expression in large, medium and small arterial vessels (aorta, renal arteries, superior mesenteric arteries) by a small animal in vivo imaging system (n =3 independent experiments). Scale bars: 50 μm. (B) Immunofluorescence detection of THSD7A protein expression in thoracic aorta after tail vein injection of adenoviral vector (n =3 independent experiments). Scale bars: 100 μm.

**Figure S2.** Endothelial knockdown of THSD7A reduces atherosclerotic plaque formation. (A-C) Representative images and quantification of THSD7A in the intima following AAV9-EC-shThsd7A mediated endothelial-specific knockdown (n = 5 mice per group). Arrows indicate the intimal layer; THSD7A (red); GFP (green); Hoechst (nuclei, blue). Scale bars: 50 μm. The green region in the vascular adventitia represents the autofluorescence of elastic fibers. (D-E) Representative images and quantification of Oil Red O stained atherosclerotic lesions in the en face aorta following endothelial THSD7A knockdown (n = 10 mice per group). Scale bars: 5 mm. (F-G) Representative images and quantification of Oil Red O stained atherosclerotic lesions in the aortic root following endothelial THSD7A knockdown (n = 10 mice per group). Scale bars: 200 μm. For panels B, C, E, and G, values are expressed as mean ± SEM. Student's t-test. *P < 0.05, **P < 0.01. n.s: not significant.

**Figure S3.** Cell type annotations and relative expression levels of *THSD7A* in each cell type. (A) Marker genes specifically expressed in different cell types. (B) Relative expression levels of *THSD7A* in different cell clusters; *THSD7A* is primarily expressed and localized in endothelial cells.

**Figure S4.** Bright-field images of cell adhesion in mouse monocyte-endothelial cells following THSD7A overexpression. Scale bars: 50 μm.

**Figure S5.** Adenovirus vector-mediated overexpression of THSD7A. (A, C) Expression of GFP in HUVECs and HCAECs at different adenovirus multiplicity of infection (MOI) levels. Scale bars: 50 μm. (B, D) Representative images and quantification of THSD7A protein expression levels in HUVECs and HCAECs following adenovirus transfection (MOI: 10) (n = 3 independent experiments). Values are expressed as mean ± SEM. Student’s t-test. *p < 0.05.

**Figure S6.** Overexpression of THSD7A promotes monocyte-endothelial cell adhesion. (A) Fluorescence and bright-field images of monocyte-endothelial cell adhesion in HUVECs following THSD7A overexpression. Scale bars: 100 μm. (B) Fluorescence and bright-field images of monocyte-endothelial cell adhesion in HCAECs following THSD7A overexpression. Scale bars: 100 μm.

**Figure S7.** ox-LDL increases endothelial cell apoptosis. (A-B) Representative images and quantification of apoptotic cells in HUVECs and HCAECs following 24 h treatment with 100 μg/mL ox-LDL, as determined by TUNEL staining (n = 3 independent experiments). Scale bars: 20 μm. Values are expressed as mean ± SEM. Unpaired two-tailed Student’s t-test. *p < 0.05. n.s: not significant.

**Figure S8.** ox-LDL induces endothelial inflammation. (A-D) mRNA expression levels of inflammatory markers (*ICAM1*, *VCAM1*, *SELE*, and *IL6*) in HUVECs and HCAECs following treatment with 100 μg/mL ox-LDL for 24 h (n = 3 independent experiments). (E-G) Representative images and quantification of monocyte-endothelial cell adhesion following treatment with 100 μg/mL ox-LDL for 24 h (n = 3 independent experiments). Scale bars: 100 μm. Values are expressed as mean ± SEM. Unpaired two-tailed Student’s t-test. **p < 0.01, ***p < 0.001.

**Figure S9.** Recombinant protein THSD7A promotes endothelial cell inflammation. (A-D, I-L) Expression of *ICAM1*, *VCAM1*, *SELE*, and *IL6* in HUVECs or HCAECs treated with rhTHSD7A for 24 h (n = 3 independent experiments). Values are presented as mean ± SEM. Student’s t-test. *p < 0.05, **p < 0.01. (E-F, M-N) Representative images and quantification of cell adhesion in HUVECs or HCAECs treated with rhTHSD7A for 24 h (n = 3 independent experiments). Scale bars: 100 μm. Values are expressed as mean ± SEM. Student’s t-test. ***p < 0.001. (G-H, O-P) Expression of *CEBPD* and *IL1A* in HUVECs or HCAECs treated with rhTHSD7A for 24 h (n = 3 independent experiments). Values are expressed as mean ± SEM. Student’s t-test. *p < 0.05, **p < 0.01.

**Figure S10.** The role of THSD7A in paracrine signaling. PA: Patient-matched proximal adjacent tissue; AS: atherosclerotic plaque (n = 3 per group). (A, E) Cell clustering of integrins *αvβ3* (*ITGAV* and *ITGB3*) in single-cell transcriptomics. (B-D, F-H) Violin plots showing expression levels of integrins *ITGAV* and *ITGB3* in endothelial cells, macrophages, and smooth muscle cells within carotid atherosclerotic plaques. (I-J) Expression of the cell proliferation markers *MKI67* and *PCNA* following treatment of smooth muscle cells with recombinant THSD7A (rhTHSD7A) (n = 3 independent experiments). (K-L) Expression of the cell migration markers *MMP9* and *MMP2* following treatment of smooth muscle cells with recombinant THSD7A (n = 3 independent experiments). (M-N) Expression of apoptosis markers *BCL2* and *BAX* in smooth muscle cells following treatment with recombinant THSD7A (n = 3 independent experiments). (O-P) Expression of inflammatory markers *TNF-α* and *IL1β* in macrophages following treatment with recombinant THSD7A (n = 3 independent experiments). For panels B, C, D, F, G, H, I, J, K, L, M, N, O and P, values are expressed as mean ± SEM. Student’s t-test. *p < 0.05, **p < 0.01, ***p < 0.001. n.s: not significant.
